# Inetetamab, a novel anti-HER2 monoclonal antibody, exhibits potent synergistic anticancer effects with cisplatin by inducing pyroptosis in lung adenocarcinoma

**DOI:** 10.7150/ijbs.82980

**Published:** 2023-08-06

**Authors:** Jinfang Cui, Yuchao He, Fuyi Zhu, Wenchen Gong, Ran Zuo, Yu Wang, Yi Luo, Liwei Chen, Chengmeng Wang, Gengwei Huo, Hailing Lu, Zhiyong Liu, Peng Chen, Hua Guo

**Affiliations:** 1 Department of Tumor Cell Biology, Tianjin Medical University Cancer Institute and Hospital, Tianjin 300060, China.; 2 Department of Thoracic Oncology, Lung Cancer Diagnosis and Treatment Center, Tianjin Medical University Cancer Institute and Hospital, Tianjin 300060, China.; 3 National Clinical Research Center for Cancer, Key Laboratory of Cancer Prevention and Therapy, Tianjin's Clinical Research Center for Cancer, Tianjin 300060, China.; 4 Department of Pathology, Tianjin Medical University Cancer Institute and Hospital, Tianjin 300060, China.; 5 Department of Oncology, First Hospital of Harbin Medical University, Harbin, 150000, China.

**Keywords:** anti-HER2 monoclonal antibody, lung adenocarcinoma, cisplatin, HER2/AKT/Nrf2 signaling pathway, pyroptosis

## Abstract

Cisplatin is a first-line chemotherapy drug for lung adenocarcinoma (LUAD). However, its therapeutic efficacy is limited because of serious side effects and acquired drug resistance. Targeting HER2 has been proven to be a viable therapeutic strategy against LUAD. Moreover, inetetamab, an innovative anti-HER2 monoclonal antibody, has a more potent antibody-dependent cell-mediated cytotoxicity (ADCC)-inducing effect than trastuzumab, which has been shown to be an effective and rational strategy in the clinic when combined with multiple chemotherapeutic agents. Thus, the present study aimed to explore the synergistic effects of cisplatin (DDP) and inetetamab in LUAD cells and investigate the detailed underlying mechanisms.

Here, *in vitro* and *in vivo*, we found that the combination of inetetamab and cisplatin induced synergistic effects, including induction of pyroptosis, in LUAD. Mechanistic studies revealed that inetetamab combined with cisplatin inhibited HER2/AKT/Nrf2 signaling to increase ROS levels, which triggered NLRP3/caspase-1/GSDMB-mediated pyroptosis to synergistically enhance antitumor efficacy in LUAD cells. In addition, cisplatin enhanced the PBMC-killing ability of inetetamab by inducing GSDMB-mediated pyroptosis, which can be explained by increased secretion of IFN-γ.

Our study reveals that the anti-HER2 monoclonal antibody inetetamab may be an attractive candidate for LUAD therapy, which opens new avenues for therapeutic interventions for LUAD.

## Introduction

Considering that lung adenocarcinoma (LUAD) is usually diagnosed in the late stage, most patients need systemic chemotherapy^1^. Platinum compounds, such as cisplatin, are front-line chemotherapeutic agents for LUAD^2^. However, serious side effects and acquired resistance to chemotherapeutic agents have created various problems in the clinical optimization of chemotherapy^3^. A large body of evidence shows that the mechanism underlying cisplatin resistance is complex and multifactorial^4^, ^5^. The currently used platinum-based chemotherapy combination strategies are far from perfect for LUAD patients because of increased adverse effects and limited efficacy^6^, ^7^. Thus, new agents to overcome cisplatin resistance and reduce adverse effects to improve the effect of LUAD treatment are desperately needed.

HER2 is a member of the family of ErbB tyrosine kinase receptors, which mediate tumor progression^8^. Moreover, HER2 amplification and overexpression have been observed in several types of human cancer, including LUAD^9^. Trastuzumab, the first therapeutic developed to target HER2, can bind to extracellular domain IV of HER2 and exert multiple antitumor effects by inhibiting downstream AKT or ERK1/2 signaling and activating antibody-dependent cell-mediated cytotoxicity (ADCC)^10^. The addition of trastuzumab to chemotherapy could significantly enhance the antitumor effect in various cancer types, including lung cancer^11^, ^12^. However, challenges such as drug shortages, cost, drug resistance and various side effects persist; therefore, biosimilars that possess the advantages of low cost and good accessibility^13^ are being pursued with great interest^14^, ^15^. Recently, a trastuzumab biosimilar, inetetamab, was developed by Shanghai CP Guojian Pharmaceutical Co. in China^16^. Inetetamab is an innovative anti-HER2 monoclonal antibody developed and first marketed in China that has been approved for the treatment of HER2-positive advanced metastatic breast cancer. Furthermore, inetetamab with amino acid modification of the Fc region has a more potent ADCC effect than trastuzumab (https://tbcr.amegroups.com/article/view/61051/html), which plays a key role in the antitumor activity of anti-HER2 monoclonal antibodies^16^. Inetetamab combined with vinorelbine has achieved significant efficacy and good safety in metastatic breast cancer^16^. However, there is no report on the combined use of inetetamab and chemotherapy drugs in lung cancer.

Therefore, this study investigated whether the addition of inetetamab might enhance the antitumorigenic effects of cisplatin on LUAD and uncovered the potential underlying mechanisms. Hence, this study will broaden our knowledge in this field and provide potential therapeutic agents for LUAD treatment in the clinic.

## Materials and Methods

### Bioinformatics analysis

Herein, we used web-based tools available on the GEPIA (Gene Expression Profiling Interactive Analysis) website, which contains data from The Cancer Genome Atlas (TCGA) and Genotype-Tissues Expression (GTEx) databases, to detect HER2, caspase-1, and GSDMB mRNA levels in LUAD^17^.

### Cell lines and reagents

The human lung adenocarcinoma cell lines PC9, Calu3, H1299, A549, and H1975 and the HER2-positive human breast cancer SKBR3 cell line were maintained in our laboratory, as previously reported 18, 19. The A549/DDP (cisplatin-resistant A549 cell line) cell line was a gracious gift from Dr. Lu of Harbin Medical University^20^.

All cells were cultured in RPMI 1640 media (Cat# 31800-022, Gibco, USA) with 10% fetal bovine serum (Cat# SH30109.02, HyClone, USA) and 1% penicillin/streptomycin (Cat# 15140-148, Gibco, USA). The cells were maintained at 37 °C in a humidified incubator with 5% CO_2_. All cells were periodically authenticated using short tandem repeat (STR) DNA profiling and then tested to confirm that they were mycoplasma contamination-free^21^.

In addition, the trastuzumab biosimilar product (Inetetamab, also known as Cipterbin®, Cat# 202205018) was developed and supplied by Shanghai CP Guojian Pharmaceutical Co. in China. Moreover, cisplatin was obtained from Hansoh Pharmaceutical Co. Ltd. (Cat# 601230101, Jiangsu, China). For the intervention experiment, cells were subsequently preincubated with 50 μM Z-VAD-FMK (Cat# HY-16658, MedChemExpress, NY, USA), 10 ng/ml IFN-γ (Cat# RP01038, ABclonal), or 5 mM N-acetylcysteine (NAC, Cat# 616-91-1, SIGMA, Shanghai, China) for 24 hours before cisplatin and inetetamab treatment.

### Retroviral infection and transfection

The shRNA HER2 target sequence was 5′-GCCTTCGACAACCTCTATTAC-3′^22^. Then, A549/DDP cells were infected with lentiviral particles and cultured in complete RPMI 1640 medium containing puromycin (Santa Cruz Biotechnology, Santa Cruz, CA) to select HER2-silenced cell clones. Finally, cells transfected with scrambled shRNA were used as controls.

### Western blot analysis

Western blot analysis was performed according to a previously described standard method^23^. Primary antibodies against the following targets were used for the Western blot analyses: HER2 (#2165, diluted at 1: 500, Cell Signaling Technology), Cyclin B1 (#4138, 1: 1000, Cell Signaling Technology), Cyclin D1 (#2922, 1: 1000, Cell Signaling Technology), HMGB1 (ab79823, 1: 10000, Abcam), GSDMB (A7474, 1: 1000, ABclonal), caspase-1 (A18646, 1: 1000, ABclonal), NLRP3 (A12694, 1: 1000, ABclonal), ERK (#9102, 1: 1000, Cell Signaling Technology), phosphorylated (p)-ERK (#4370, 1: 1000, Cell Signaling Technology), AKT (#9272, 1: 1000, Cell Signaling Technology), phosphorylated (p)-AKT (#4060, diluted at 1: 1000, Cell Signaling Technology), Nrf2 (#12721, 1: 1000, Cell Signaling Technology), β-actin (used as the loading control; sc-47778, Santa Cruz Biotechnology), GAPDH (used as the loading control; sc-47724, Santa Cruz Biotechnology), and β-tubulin (used as the loading control; #2128, Cell Signaling Technology).

### Combination Index

The combination index (CI) was calculated to evaluate the efficacy of the combination treatment of inetetamab and cisplatin based on the median dose-effect analysis by Chou and Talalay^24^. Furthermore, CI analysis was performed using CompuSyn Software (ComboSyn, Inc., Paramus, NJ, USA). The combined effect is indicated as follows: CI < 1 means synergism; CI = 1 indicates additive effects; and CI > 1 denotes an antagonistic effect.

### Cell viability assay, colony formation assay, flow cytometric analysis of cell cycle distribution

Cell viability assays, colony formation assays, and flow cytometric analysis of cell cycle distribution were performed as described previously^23^. Alternatively, cell proliferation was measured with an EdU incorporation assay using the BeyoClick™ EdU-594 Cell Proliferation Kit (Cat# C0071S, Beyotime Biotech. Inc.) following the manufacturer's instructions. Briefly, cells were seeded in 48-well plates (5 × 10^3^ cells per well) and incubated for 48 hours with cisplatin and/or inetetamab. Cells were then treated with EdU (10 μM) for 2 h at 37 °C and Hoechst 33342 was used for nuclear staining. Finally, images were acquired using a fluorescence microscope.

### Immunofluorescence

Herein, cells were seeded in twelve-well plates and fixed in 4% paraformaldehyde (Cat# P0099, Beyotime Biotech. Inc.)^23^. Subsequently, the cells were blocked with 10% bovine serum albumin for 1 hour at room temperature and incubated with primary antibody. This was followed by incubation with Alexa Fluor-conjugated secondary antibodies (Cat# A21207, Invitrogen). Afterward, the nuclei were counterstained with 4′,6-diamidino-2-phenylindole (DAPI, Cat# D9542, Sigma), and the cells were then viewed with a fluorescence microscope.

### Microscopic imaging

Cells (1.0 × 10^4^ cells/well) were seeded into 12-well plates to observe the morphological characteristics of pyroptosis. Static bright-field images were captured using an inverted microscope (EVOS™ M5000, ThermoFisher) or by scanning electron microscopy (SEM) at room temperature^25^. All image data shown are representative of at least three randomly selected fields.

### Lactate dehydrogenase (LDH) release assay

Pyroptosis was measured based on the level of secreted LDH in the supernatants of treated cells with an LDH cytotoxicity assay kit (Cat# C0017, Beyotime Biotech. Inc.) following the manufacturer's instructions^25^.

### Flow cytometric analysis of cell pyroptosis

Flow cytometry analysis of pyroptotic cell death was performed using an Annexin V-FITC/PI staining kit (Cat# 556547, BD Pharmingen) or the Annexin V-PE/7-AAD Apoptosis Detection Kit (Cat# 559763, BD Pharmingen) by following the manufacturer's instructions. In addition, H1299, A549/DDP, and H1975 cells were treated with specific concentrations of cisplatin and/or inetetamab for 48 hours. Afterward, the cells were collected, washed with PBS twice, and stained. Subsequently, samples were analyzed on the FACS Aria flow cytometer, and data were processed in the software Flow Jo. Annexin-V can stain pyroptotic cells because membrane rupture allows for Annexin-V recognition of phosphatidylserine on the inner leaflet of the plasma membrane. Referring to the literature, Annexin-V- and PI- or 7-AAD-positive cells were considered pyroptotic cells^26^.

### Enzyme-linked immunosorbent assay (ELISA)

After the designated treatment, human high mobility group protein B1 (HMGB1) and interferon-gamma (IFN-γ) concentrations in the cell supernatant were detected using commercial kits (Cat# P09429, Cat#P01579, CUSABIO Biotech) based on the recommended protocol in the manufacturer's instructions 25. The measurements were acquired with a microplate reader (BioTek, Winooski, VT, USA) at 450 nm.

### Reactive oxygen species (ROS) measurement

In the current study, intracellular ROS measurement was performed as previously described by a fluorescence microplate reader^27^, ^28^. Briefly, H1299, A549/DDP, and H1975 cells were seeded in a 96-well plate and incubated with cisplatin and/or inetetamab in the presence or absence of NAC for 48 hours. After washing, the cells were stained with 10 μM 2,7-dichlorofluorescein-diacetate (DCFH-DA) at 37 °C for 30 min by following the manufacturer's instructions (CA1410, Solarbio, Beijing, China). Moreover, green fluorescence was observed under a fluorescence microscope at excitation and emission wavelengths of 488 and 525 nm, respectively, and the fluorophore signal was recorded in a microplate reader.

### Preparation of human PBMCs

Peripheral blood mononuclear cells (PBMCs) were isolated from fresh heparinized whole blood of healthy donors using Lymphoprep density gradient centrifugation (Cat# 07801 StemCell Technologies, Inc.) with their consent and ethics approval. Briefly, whole blood was collected from healthy human donors and diluted by half with PBS. Afterward, the blood was layered onto 15 mL of lymphocyte separation medium. Subsequently, samples were centrifuged at 800 ×g for 20 min, and the PBMC interface was aspirated with a pipette tip and washed in 40 mL of PBS by centrifugation. Moreover, PBMCs were resuspended in 1640 complete culture medium plated and preincubated at 37 °C for up to 18 hours in the presence of human interleukin-2 (IL-2, 800 U/ml, Cat# 78036 StemCell)^29^.

### PBMC killing assays

Target cells (4 × 10^3^) were seeded in 96-well flat-bottomed plates with 100 μl of medium per well to assay killing by PBMCs. After the cells adhered, they were incubated with 4 μM calcein AM (Cat# C2012, Beyotime Biotech. Inc.) in serum-free medium for 30 min at 37 °C in the dark based on the manufacturer's protocol 30. The cells were then washed twice with phosphate-buffered saline (PBS) and treated with cisplatin and/or inetetamab. Then, PBMCs were subsequently added to the target cells for 6 hours at a 50:1 effector/target cell (E/T) ratio in 96-well plates.

Intact target cells not cocultured with PBMCs were used to determine the spontaneous and maximal release of calcein. The fluorescence of the released calcein was measured with a fluorescence microplate reader at excitation and emission wavelengths of 494 nm and 514 nm, respectively. Furthermore, the percentage of calcein release indicating target cell death was calculated as follows: (experimental - spontaneous release)/(maximum - spontaneous release) × 100%. Calcein AM is a cell-permeable green fluorescent probe that is hydrolyzed by endogenous cellular esterases to produce calcein, which fluoresces and is retained in the cytoplasm.

### Construction of pyroptosis regulator phenotypes

Herein, we summarized data on 28 pyroptosis-related molecules from existing studies^31^, ^32^: GSDMA, GSDMB, GSDMC, GSDMD, DFNB59, ZBP1, PYCARD, PRF1, NLRP1, NLRP3, NLRC4, NLRP9, NAIP, IL1B, GZMA, GZMB, DHX9, DFNA5, DDX3X, CTSG, CASP1, CASP4, CASP5, CASP6, CASP8, APIP, and AIM2. First, unsupervised clustering analysis (K-Means, based on Euclidean distance) was used to identify the pyroptosis phenotypes in LUAD cancer based on the expression levels of 28 pyroptosis-related genes to classify patients. In addition, the optimal clustering number of the LUAD cohort was determined by the consensus clustering algorithm, and its stability was further verified. Moreover, the R package 'ConsensusClusterPlus' was used in the process 32.

### Correlation with drug sensitivity

Genomics of Drug Sensitivity in Cancer (GDSC, https://www.cancerrxgene.org/) a publicly available pharmacogenomics database, was used to measure cisplatin sensitivity. Moreover, the R package 'PRRophetic'^32^ was used for prediction, and the ridge regression model was constructed using the 'linearRidge' function of the R package 'Ridge' to determine the IC50 of cisplatin in LUAD samples to indicate drug sensitivity.

### Xenograft models

Animal experiments were approved by the Ethics Committee of Tianjin Medical University, China (permit no. SYXK2023-0001). The experimental process was previously described^33^. A total of 5 × 10^6^ H1975 cells that had been resuspended in 100 μL PBS were injected subcutaneously into the right flanks of female BALB/c nude mice (4 weeks old, 17±3 g weight, Jiangsu GemPharmatech Co., Ltd.). Tumor volume (V) was calculated with the following formula: length × width^2^ × 1/2. The mice bearing H1975 cell-derived tumors were randomly divided into four groups (n = 6 per group) when the tumor volume reached approximately 100 mm^3^. (1) Control group: mice were injected with phosphate-buffered saline (PBS). (2) Cisplatin group: mice were injected with 2.5 mg/kg cisplatin intraperitoneally once a week. (3) Inetetamab group: mice were injected with 15 mg/kg inetetamab intraperitoneally once a week. (4) Combination group: both cisplatin and inetetamab were administered according to the aforementioned regimens. The tumor size was checked with a caliper, and the variation in the weight of the mice was recorded by a scale. Mice were monitored every 3 days after injection for a total of 37 days. Tumor weight was measured after excision on day 37.

### Tissue specimens and immunohistochemistry

Xenograft specimens derived from the H1975 cell line were formalin fixed and paraffin embedded. Tissue microarrays of LUAD tissues (TFLungade-01, n=90) from 45 cisplatin-sensitive and 45 cisplatin-resistant patients were purchased from Shanghai Tufei Biotech^34^. The cisplatin-resistant group included patients with disease progression or stable disease without extended (6 months) progression-free survival (PFS), and the cisplatin-sensitive group included patients with a complete or partial response or stable disease with prolonged PFS (≥ 6 months)^35^. The study protocol was carefully explained to the participants, and written informed consent was obtained from all participants. Ethical clearance and approval (No. bc2023053) were obtained from the Ethics Committee of Tianjin Medical University Cancer Institute and Hospital.

Immunohistochemistry (IHC) staining procedures and subsequent analyses were performed as described previously^23^, ^33^. Primary antibodies against the following targets were used in the IHC experiments: HER2 (A2071, 1: 100, ABclonal), phosphorylated (p)-AKT (#4060, diluted at 1: 100, Cell Signaling Technology), Nrf2 (A0674, 1: 100, ABclonal), NLRP3 (#41768, 1: 100, Signalway Antibody), Cleaved-Caspase1 (A21296, 1: 50, ABclonal), and GSDMB (ab215729, 1: 100, Abcam). IHC results were blindly scored by two independent pathologists using the following criteria. Staining intensity was evaluated from 0 (negative) to 3 (strong). The % positive cells was classified on a 4-point scale: 0, no positive cells; 1, <30% positive cells; 2, 30%-60% positive cells; and 3, 60%-100% positive cells. The two values were multiplied together to obtain an integrated score ranging from 0 to 9 (0-1, negative; 2-3, moderate; and 4-9, strongly positive). A score ≤1 denoted low protein expression whereas a score ≥2 denoted high protein expression.

### Statistical analysis

Values are expressed as the mean ± standard deviation, and at least three independent experiments were performed if the data were quantitative. The software program GraphPad Prism 7 (San Diego, CA, USA) was used to analyze the quantitative results. Moreover, differences in continuous variables between the two groups were analyzed by two-tailed Student's tests or one-way ANOVA, and the differences in categorical variables were analyzed with χ^2^ tests. The Kaplan‒Meier method was used to generate survival curves for the subgroups of each cohort, and the log-rank (Mantel‒Cox) test was used to identify statistically significant differences. A statistically significant difference is indicated as * p < 0.05, ** p < 0.01, or *** p < 0.001.

## Results

### Inetetamab combined with cisplatin synergistically enhances antitumor efficacy in LUAD cells

To screen HER2-positive lung adenocarcinoma cell lines, Western blot analyses were performed to measure the HER2 protein levels in the LUAD cell lines, with the HER2-positive breast cancer cell line SKBR3 as a positive control. We found that HER2 expression was significantly higher in PC9, H1299, Calu3 and H1975 cell lines than in SKBR3 cells (Fig. S1A). The cisplatin-resistant cell line (A549/DDP) had higher HER2 protein expression than the cisplatin-sensitive LUAD cell line (A549) (Fig. S1B), and the HER2 expression level of A549 was comparable to that of SKBR3 (Fig. S1A). We chose H1299 with relatively low HER2 expression and H1975 with the highest HER2 expression among these four HER2-positive LUAD cell lines (Fig. S1A), as well as the A549/DDP cell line, for subsequent experiments.

**Figure 1:**
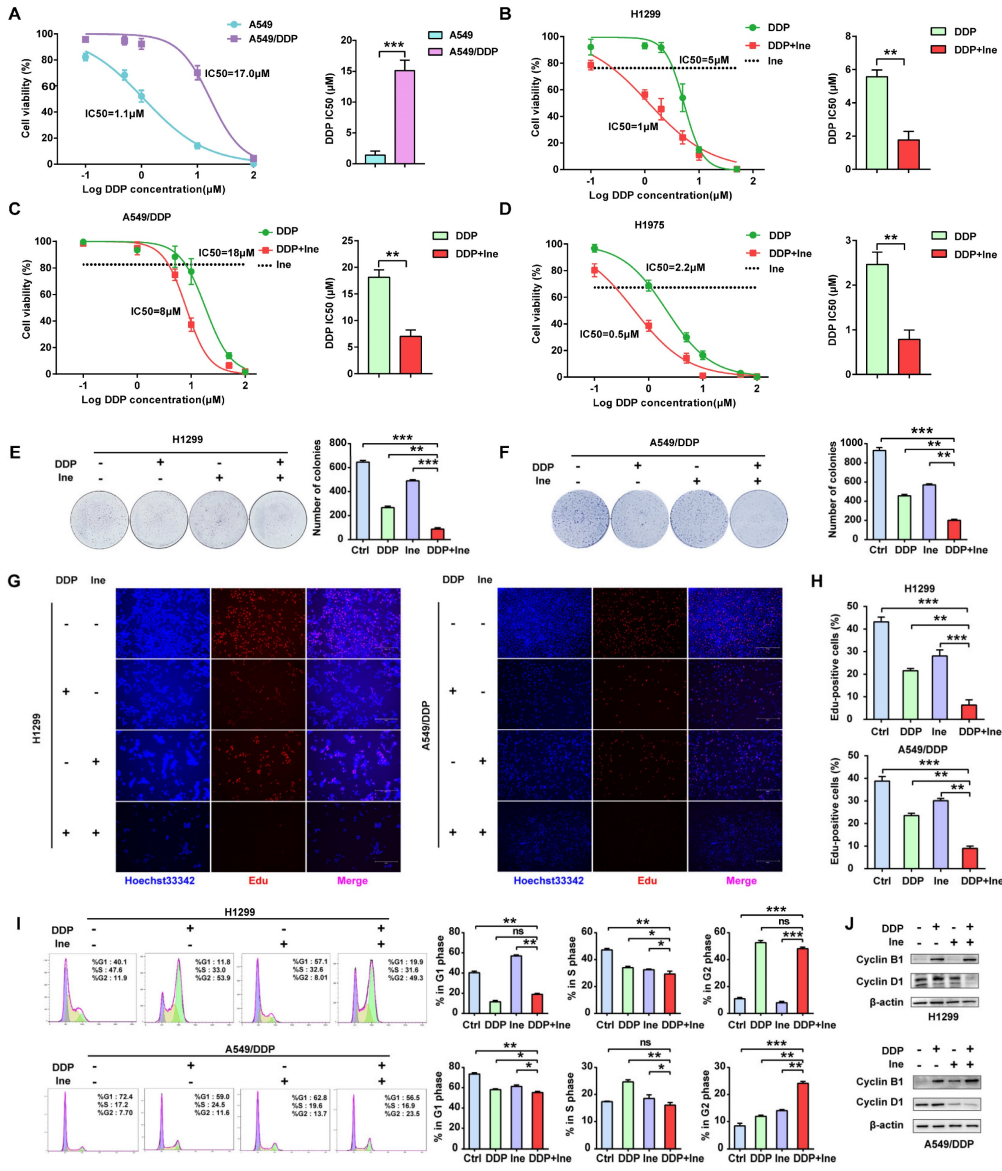
** Inetetamab synergizes with cisplatin for antitumor efficacy in LUAD cells. (A)** IC50 analysis of cisplatin sensitivity by a CCK-8 assay in A549 and A549/DDP cells. **(B)** Dose-response curves determined by the CCK-8 assay were used to calculate the IC50 values of cisplatin in the presence or absence of inetetamab in H1299. Black dotted lines indicate the cell viability at a fixed concentration of 5 μM inetetamab for 5 days. **(C)** Dose-response curves determined by the CCK-8 assay were used to calculate the IC50 values of cisplatin in the presence or absence of inetetamab in A549/DDP cells. Black dotted lines indicate the cell viability at a concentration of 10 μM inetetamab for 5 days. **(D)** Dose-response curves determined by the CCK-8 assay were used to calculate the IC50 values of cisplatin in the presence or absence of inetetamab in H1975 cells. The black dotted line indicates the same meaning as described in B. **(E)** The colony-forming efficiency of H1299 was determined. These cells were treated with DDP or Ine or a combination of both at the same time for 14 days.** (F)** The colony-forming efficiency of A549/DDP was determined. These cells were treated with DDP or Ine or a combination of both at the same time for 14 days. **(G)** Cell viability of the indicated cells treated with DDP or Ine or a combination of both was analyzed by an EdU incorporation assay at 5 days. Scale bars, 300 μm. **(H)** Quantify fraction of Edu-positive cells described in G. **(I)** The cell cycle distribution of indicated cells treated the same as in G. **(J)** Representative Western blot showing the effects of indicated drugs for 96 h on the expression levels of Cyclin B1 and Cyclin D1 in H1299 and A549/DDP cells. β-actin served as a loading control. *p < 0.05, **p < 0.01, ***p < 0.001. Ctrl, control (untreated cells); DDP, cisplatin; Ine, inetetamab

Trastuzumab can significantly enhance the antitumor effect of cisplatin in various cancer types, including lung cancer^11^, ^12^. However, whether inetetamab combined with cisplatin exerts a synergistic anticancer effect has not yet been studied. First, we verified the resistance of A549/DDP cells to cisplatin. The half-maximal inhibitory concentration (IC50) for cisplatin was dramatically increased in A549/DDP cells compared with cisplatin-sensitive cell lines (A549) (Fig. 1A). Therefore, a CCK-8 assay was applied to measure the IC50 values of cisplatin in LUAD cells in the presence or absence of inetetamab to explore whether inetetamab could synergistically enhance cisplatin-mediated cytotoxicity in LUAD cells. The results indicated that the administration of inetetamab in H1299, H1975 and A549/DDP (cisplatin-resistant cell line) cells significantly decreased the cisplatin IC50 value (Fig. 1B-D). As shown in Figure 1B, inetetamab decreased the cisplatin IC50 from 5 to 1 µM in H1299 cells. The cisplatin IC50 in A549/DDP cells also markedly decreased from 18 to 8 µM (Fig. 1C), and the cisplatin IC50 in H1975 cells decreased from 2.2 to 0.5 µM (Fig. 1D). In addition, the combination index (CI) values showed that the combination of inetetamab and cisplatin exerted synergistic cytotoxic effects at almost all tested concentrations (Fig. S2A-C). Then, 5 μM cisplatin and 5 μM inetetamab were selected for H1299 and H1975 cells, and 10 μM cisplatin and 10 μM inetetamab were selected for A549/DDP cells for subsequent experiments.

The colony formation of cells in the cisplatin combined with inetetamab group was significantly reduced compared to that in the single-agent cisplatin and inetetamab groups in both cisplatin-sensitive and cisplatin-resistant cell lines (Fig. 1E, F). EdU staining assays further demonstrated that the addition of inetetamab significantly reduced the proliferation of LUAD cells compared with that in the single-agent cisplatin group in both cisplatin-sensitive and cisplatin-resistant cell lines (Fig. 1G, H). Moreover, flow cytometry analyses demonstrated that combination treatment induced further G2/M phase arrest in A549/DDP cells and shortened the S phase of H1299 cells (Fig. 1I). Cell cycle-related protein expression was also assessed by Western blotting of cell proteins from different treatment groups. Cyclin B1, a key component in the control of cell cycle progression from G2 to M phase^36^, was upregulated under combination treatment, whereas cyclin D1, a critical regulator of G1 to S phase transition^37^, was downregulated (Fig. 1J). Therefore, these phenomena indicated that inetetamab and cisplatin synergistically inhibits the proliferation of LUAD *in vitro*.

### Inetetamab promotes cisplatin-induced pyroptosis of LUAD cells

Herein, we established a HER2 knockdown cell line with A549/DDP cells to determine whether targeting HER2 is relevant to the synergistic antitumor effects. As shown in Fig. S3A, HER2 shRNA-transfected A549/DDP cells (shHER2) displayed a decrease in the expression of HER2 compared to that in the control cells (shSCR). However, the knockdown of HER2 slightly increased the sensitivity of A549/DDP to various concentrations of cisplatin (Fig. S3B). This suggests targeting of HER2 is not the main factor in the synergistic antitumor effect of the two drugs in LUAD. The mechanism underlying cisplatin resistance is complex and multifactorial^4^, ^5^. Most often, targeting one mechanism fails to fully circumvent drug resistance^6^. In terms of the mechanism by which inetetamab exerts synergistic antitumor effects with cisplatin, we speculated that there are additional factors contributing to cisplatin sensitization apart from targeting HER2.

**Figure 2:**
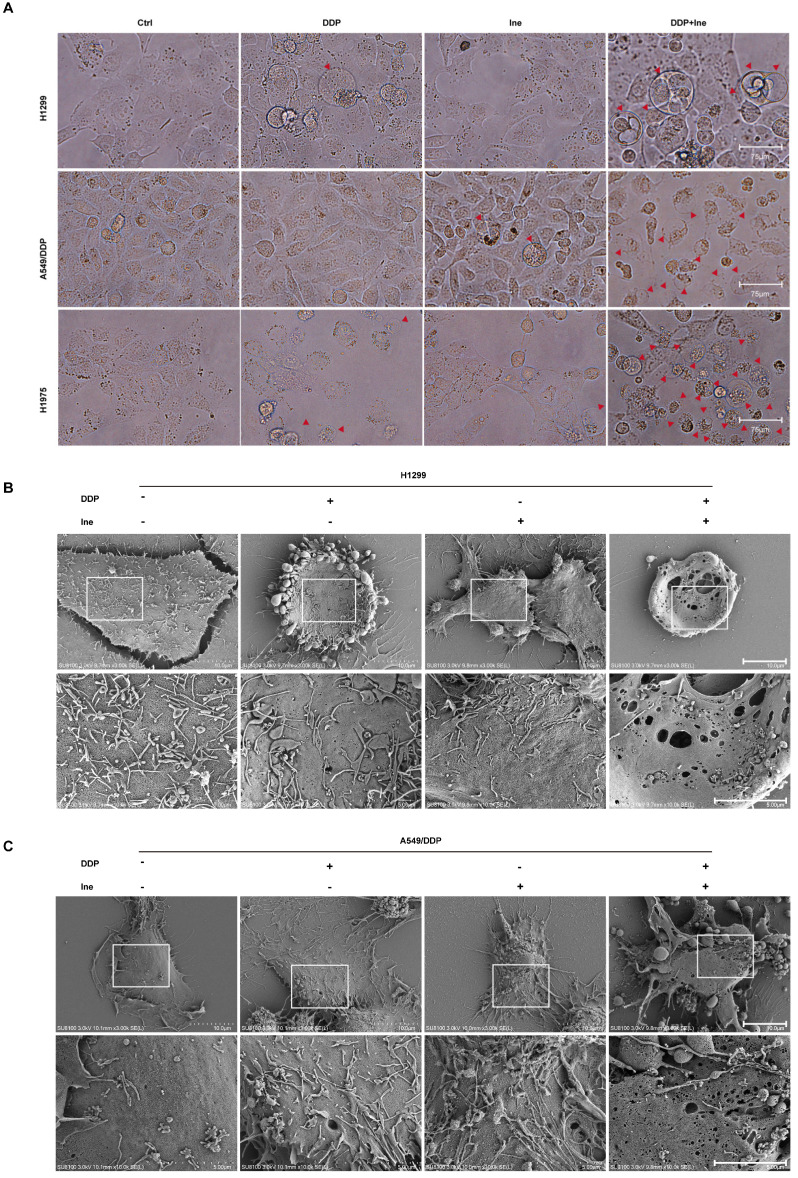
** Inetetamab promotes cisplatin-induced pyroptosis of LUAD cells. (A)** Representative microscope capture of H1299, A549/DDP and H1975 cells treated with DDP, Ine and the combination groups for 4 days. Red arrowheads indicated large bubbles emerging from the plasma membrane. Scale bar, 75 μm. **(B)** Representative transmission electronic micrographs of H1299 cells after treated with DDP, Ine and the combination for 4 days. Scale bar, 10 μm. At the bottom is the enlarged image. Scale bar, 5 μm. **(C)** Representative transmission electronic micrographs of A549/DDP cells after treated with indicated drugs. Scale bar, 10 μm. At the bottom is the enlarged image. Scale bar, 5 μm.

Therefore, the synergistic mechanism of inetetamab and cisplatin cotreatment was explored. When the cells were viewed under a light microscope, the number of typical large bubbles emerging from the plasma membrane in dying cells was highest in the two-drug combination group, and whole cells displayed swelling typical of the process (Fig. 2A). The morphological features were consistent with pyroptosis^30^, ^38^. Pyroptosis, a new type of inflammatory programmed cell death, contributes to chemosensitivity 39. Subsequently, various honeycomb pores formed on the cell membrane of combination-treated H1299 and A549/DDP cells, and the pores fused to form huge pores with a diameter greater than 1 μm, as observed by SEM (Fig. 2B, C). The formation of discrete pores in the plasma membrane is a typical feature of pyroptosis and causes water influx and cell swelling, thereby resulting in cell-membrane rupture and inflammatory cytokine release^40^.

**Figure 3:**
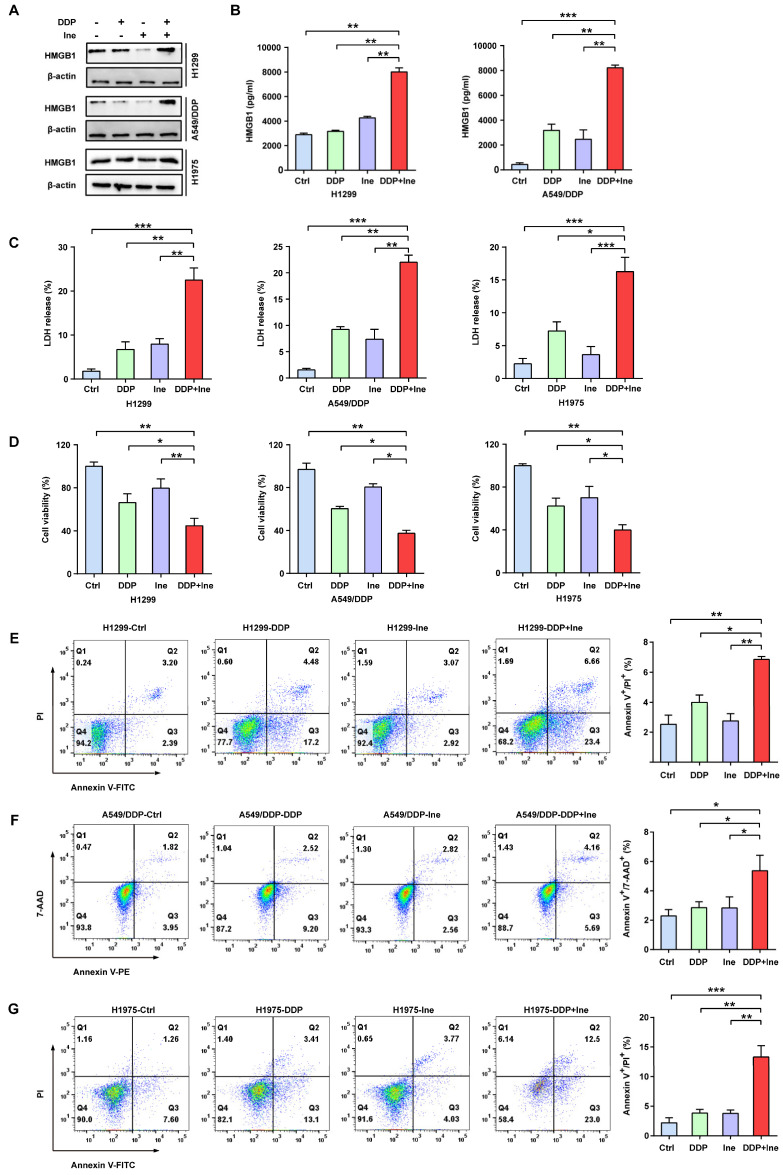
** Inetetamab increases cisplatin-induced pyroptosis of LUAD cells. (A)** The HMGB1 expression in cells treated with DDP, Ine and a combination of both for 4 days were examined by Western blot. β-actin was used as a loading control. **(B)** H1299 and A549/DDP cells were treated as described previously, and their supernatants were collected for ELISA to detect the level of HMGB1 secretion. **(C)** Cytotoxicity was detected by LDH assay. **(D)** Cell viability was measured by CCK-8 assay. **(E)** Percentage of Annexin-V/PI positive cells were measured using flow cytometry in H1299 treated with DDP, Ine and a combination of the two drugs for 4 days.** (F)** Percentage of Annexin-V/7-AAD positive cells were measured using flow cytometry in A549/DDP treated with DDP, Ine and a combination of the two drugs for 4 days. **(G)** Percentage of Annexin-V/PI positive cells were measured using flow cytometry in H1975 treated the same as in E. *p < 0.05, **p < 0.01, ***p < 0.001. Ctrl, control (untreated cells); DDP, cisplatin; Ine, inetetamab

In addition, the typical characteristics of pyroptosis were membrane pore formation, proinflammatory factor release, increased LDH release, and increased Annexin-V/PI staining according to flow cytometry^30^. HMGB1 is a main proinflammatory factor released by pyroptotic cells as a result of plasma membrane rupture and leakage^41^. Thus, we quantified the levels of HMGB1 using Western blotting and ELISA to further validate the role of pyroptosis in the combined treatment effect. We found that the HMGB1 level was significantly elevated in the combined group vs. the monotherapy groups (Fig. 3A, B), thereby indicating that the combination therapy triggered a higher degree of membrane swelling and leakage. LDH and CCK-8 analyses also showed that inetetamab combined with cisplatin significantly increased LDH release and decreased cell viability compared to that in any single-agent group (Fig. 3C, D). Furthermore, flow cytometric analysis indicated that combined drug treatment dramatically increased the proportion of Annexin-V/PI-positive cells, which suggested that the number of pyroptotic cells increased (Fig. 3E-G). The above data indicated that the pyroptosis rate in the cisplatin combined with inetetamab group of LUAD cells was higher than that in the monotherapy groups. Pharmacologically, the addition of inetetamab to cisplatin exerts antitumor effects not only by targeting HER2 but also by increasing pyroptosis, which has been proven to be a factor in cisplatin sensitization^39^.

### LUAD patients with high expression of pyroptosis-related genes are sensitive to cisplatin

**Figure 4:**
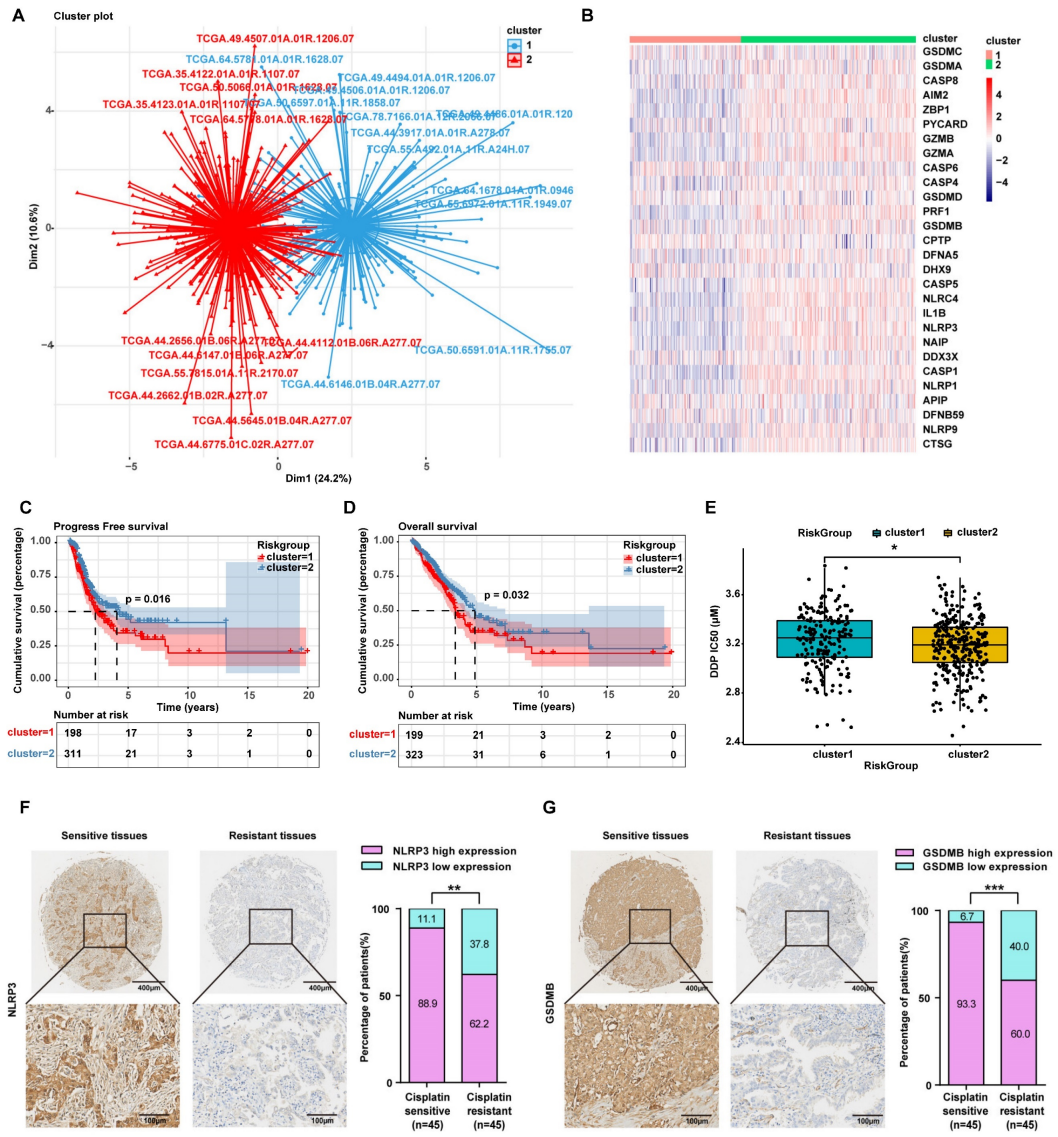
** LUAD patients with high expression of pyroptosis-related genes are sensitive to cisplatin. (A)** 522 LUAD Patients in the TCGA database were divided into two clusters by unsupervised clustering according to the expression level of pyroptosis-related genes. (Cluster2 represents LUAD patients with high expression of pyroptosis genes; cluster1 represents LUAD patients with low expression of pyroptosis genes).** (B)** Heatmap showing the differential expression of 28 pyroptosis genes signatures between the two clusters. A color key for the normalized expression data is shown at the right of the heatmap. **(C)** The Kaplan-Meier curve shows significant progress free survival (PFS) rate differences between the two kinds of pyroptosis phenotypes in the TCGA database. **(D)** The Kaplan-Meier curve shows significant overall survival (OS) rate differences between the two kinds of pyroptosis phenotypes in the TCGA database.** (E)** IC50 analysis of cisplatin between the two clusters as described in A. **(F)** Characteristic IHC images of NLRP3 in cisplatin-sensitive and cisplatin-resistant LUAD tissues from the cisplatin-sensitive group (PFS ≥ 6 months) and the cisplatin-resistant group (PFS < 6 months). The percentages of patients with high expression and low expression of NLRP3 were assigned according to different responses to cisplatin (right panel). **(G)** Characteristic IHC images of GSDMB in cisplatin-sensitive and cisplatin-resistant LUAD tissues from the cisplatin-sensitive group (PFS ≥ 6 months) and the cisplatin-resistant group (PFS < 6 months). The percentages of patients with high expression and low expression of GSDMB were assigned according to different responses to cisplatin (right panel). *p < 0.05, **p < 0.01, ***p < 0.001.

Herein, to further confirm that the mechanism by which inetetamab combined with cisplatin exerts a synergistic antitumor effect involves the induction of LUAD cell pyroptosis, we divided 522 LUAD patients in the TCGA database into two groups by unsupervised clustering based on the expression levels of pyroptosis-related genes (Fig. 4A, B). We found that the group with high expression of pyroptosis-related genes, including GSDMB, had longer OS and PFS (Fig. 4C, D) and was more sensitive to cisplatin (Fig. 4E).

To explore whether there is a relationship between pyroptosis-related protein expression and cisplatin sensitivity in the clinic, we assessed the expression level of NLRP3 and GSDMB in cisplatin-sensitive and -resistant LUAD tissues by performing an IHC analysis of a tissue microarray containing 90 LUAD tissue samples which were collected from LUAD patients who had been treated with cisplatin. IHC assays showed that the NLRP3 and GSDMB expression levels were higher in the cisplatin-sensitive group (PFS ≥ 6 months) than in the cisplatin-resistant group (PFS < 6 months, Fig. 4F, G), suggesting that high pyroptosis-related protein (NLRP3 and GSDMB) expression in clinical LUAD specimens is significantly associated with chemosensitivity. These results further suggested that pyroptosis can sensitize cells to cisplatin and even reverse cisplatin resistance, and thus, patients with high pyroptosis-related gene expression have a better prognosis.

### Inetetamab combined with cisplatin triggers pyroptosis via the caspase-1/GSDMB axis

**Figure 5:**
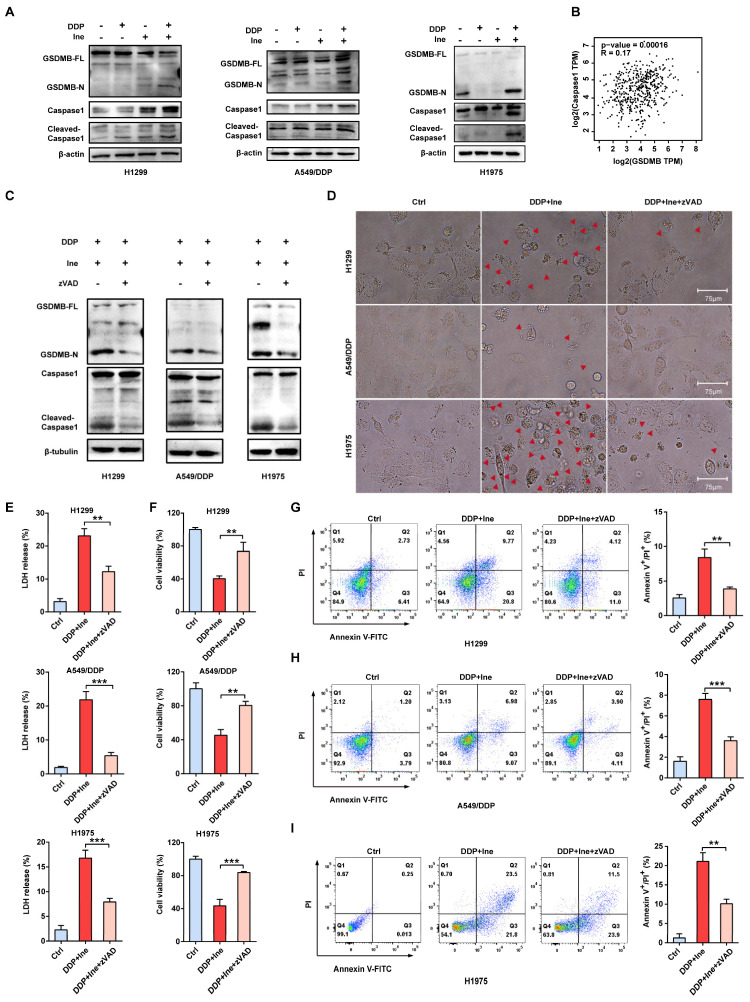
** Inetetamab combined with cisplatin triggers pyroptosis via the caspase-1/GSDMB axis. (A)** Western blot analysis of the cleavage of GSDMB and caspase-1 in DDP, Ine and a combination of both treated H1299, A549/DDP and H1975 cells for 4 days. β-actin and β-tubulin served as a loading control. **(B)** The correlation between GSDMB and caspase-1 mRNA expression in LUAD was identified by the TCGA database.** (C)** Western blot analysis of the cleavage of GSDMB and caspase-1 in H1299, A549/DDP and H1975 cells treated with a combination of DDP and Ine for 4 days with or without pretreatment of Z-VAD-FMK (50 μM). **(D)** The features of cell pyroptosis were detected at 48h after DDP and Ine cotreatment in these indicated cells with or without pretreatment of Z-VAD-FMK. Red arrowheads indicated large bubbles emerging from the plasma membrane. Scale bar, 75 μm. **(E)** LDH release assay was performed to characterize cytotoxicity in indicated cells treated as described in C. **(F)** CCK-8 assay was performed to detect cell viability in indicated cells treated as described in C. **(G)** Percentage of Annexin-V and PI or 7-AAD positive cells were measured using flow cytometry in H1299 treated with a combination of DDP and Ine for 4 days with or without pretreatment of Z-VAD-FMK (50 μM). **(H)** Percentage of Annexin-V/PI positive cells were measured using flow cytometry in A549/DDP treated with a combination of DDP and Ine for 4 days with or without pretreatment of Z-VAD-FMK (50 μM).** (I)** Percentage of Annexin-V and PI or 7-AAD positive cells were measured using flow cytometry in H1975 treated with a combination of DDP and Ine for 4 days with or without pretreatment of Z-VAD-FMK (50 μM). *p < 0.05, **p < 0.01, ***p < 0.001. Ctrl, control (untreated cells); DDP, cisplatin; Ine, inetetamab; zVAD, Z-VAD-FMK; GSDMB-N, GSDMB N-terminus; GSDMB-FL, full-length GSDMB

The signature terminal events of pyroptosis are the activation of inflammatory caspases and the release of the GSDM N-terminus to form pores in the plasma membrane^38^. Recently, GSDMB has been demonstrated to be cleaved by granzyme A from cytotoxic lymphocytes to induce pyroptosis in GSDMB-expressing tumor cells^30^. GSDMB is cleaved by caspase-1 at site aspartate 236 to trigger pyroptosis in human embryonic kidney (HEK) 293T cells^42^. In our Western blot analysis, the levels of N-terminal GSDMB and cleaved caspase-1 were elevated in combination-treated H1299, A549/DDP, and H1975 cells compared to the cisplatin monotherapy group and the inetetamab monotherapy group (Fig. 5A). Cleaved caspase-1 was also increased in the inetetamab group and the cisplatin group alone compared with the blank control group (Fig. 5A). Considering the literature and our experimental results suggesting that the caspase-1/GSDMB axis plays an important role in triggering pyroptosis, we analyzed the expression profiles of the GSDMB and caspase-1 genes in LUAD tumor tissues in the GEPIA database. The results demonstrated that the mRNA level of GSDMB was positively correlated with the expression level of caspase-1 (Fig. 5B). Thus, H1299, A549/DDP, and H1975 cells were pretreated with the caspase-1 inhibitor Z-VAD-FMK for 24 hours and then treated with inetetamab combined with cisplatin to corroborate these observations. We found that adding the inhibitor suppressed the release of N-GSDMB (Fig. 5C), decreased the pyroptosis ratio and suppressed the development of pyroptosis-like features in cells (Fig. 5D). Our results also indicated that Z-VAD-FMK treatment inhibited LDH release (Fig. 5E) and increased cell viability (Fig. 5F). Furthermore, flow cytometry showed that inhibitor treatment reduced the ratio of Annexin V^+^/PI^+^ cells (Fig. 5G-I). The above results revealed that inetetamab combined with cisplatin triggered pyroptosis by activating the caspase-1/GSDMB axis, which is one of the contributors to the enhancement of cisplatin sensitivity and reversal of cisplatin resistance by inetetamab in LUAD cells.

### Inetetamab combined with cisplatin activates caspase-1 via HER2/AKT/Nrf2 signaling-triggered ROS accumulation

**Figure 6:**
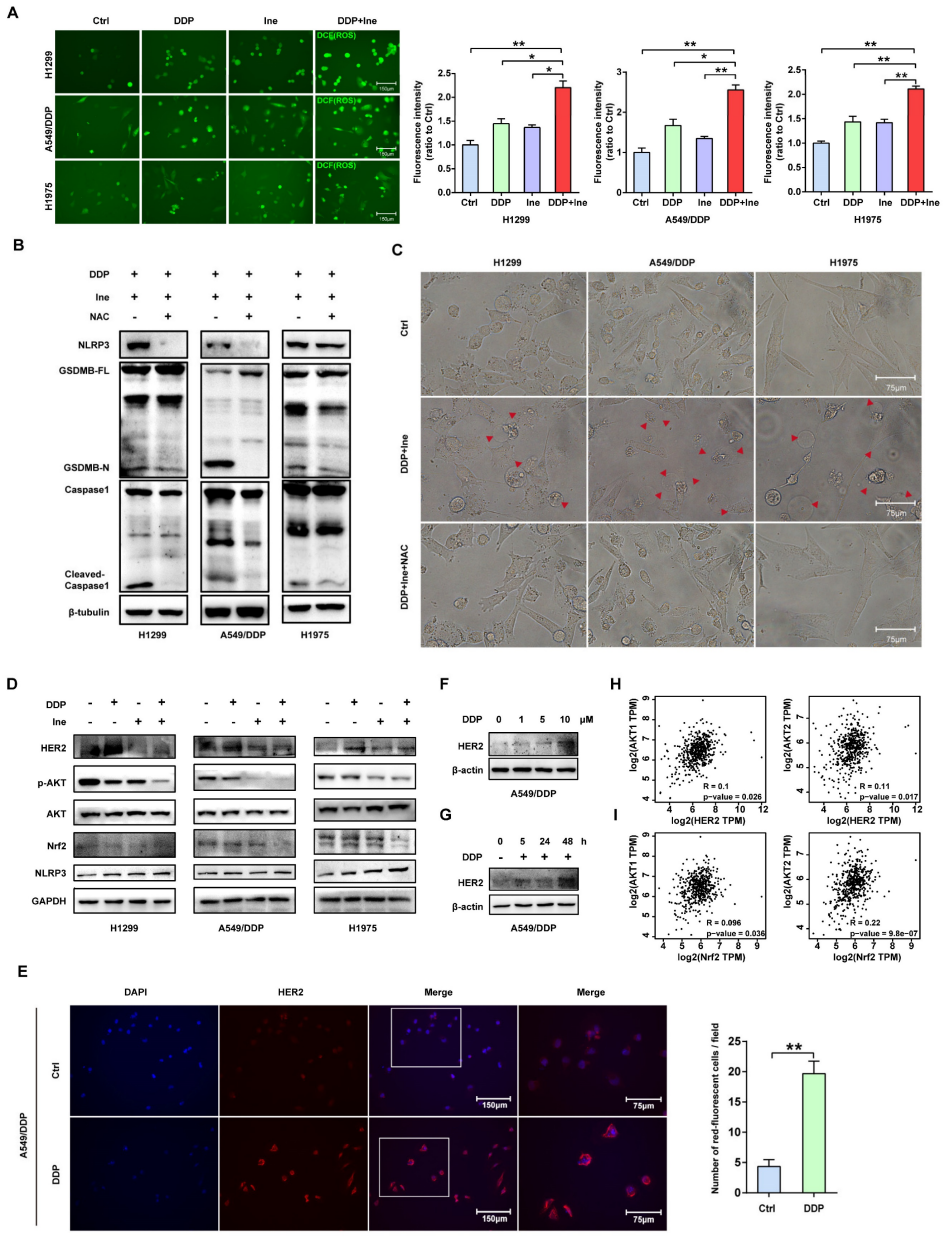
** Inetetamab combined with cisplatin activates caspase-1 via HER2/AKT/Nrf2 signaling-triggered ROS accumulation. (A)** The ROS generated after H1299, A549/DDP and H1975 cells were treated with DDP and Ine alone or in combination. The bar graph on the right is the quantitative analysis of ROS immunofluorescence. Notes: ROS levels were detected by dichlorofluorescein (DCF) fluorescence intensity. **(B)** Western blot analysis of the cleavage of GSDMB and caspase-1 in H1299, A549/DDP and H1975 cells treated with a combination of DDP and Ine for 4 days with or without pretreatment of NAC (5 mM). **(C)** Representative microscope captures of H1299, A549/DDP and H1975 cells treated as described in B. Red arrowheads indicated large bubbles emerging from the plasma membrane. Scale bar, 75 μm. **(D)** Western blot analysis of key signal transduction proteins in indicated cells treated with DDP and Ine alone or in combination for 4 days. GAPDH served as a loading control. **(E)** Immunofluorescent staining confirmed the HER2 expression of A549/DDP treated with or without cisplatin. Scale bar, 150 μm. Pictures at higher magnification are shown. Scale bar, 75 μm. The bar graph on the right is the quantification of the red-fluorescent cells.** (F)** Western blot analysis of HER2 expression in A549/DDP at various concentrations of cisplatin (0 μM, 1 μM, 5 μM, 10 μM) for 4 days. GAPDH served as a loading control. **(G)** Western blot analysis of HER2 expression in A549/DDP treated with cisplatin (10 μM) at different time (0 h, 5 h, 24 h, 48 h). GAPDH served as a loading control. **(H)** The correlation between HER2 and AKT1 and AKT2 mRNA expression in LUAD was identified by the TCGA database. **(I)** The correlation between Nrf2 and AKT1 and AKT2 mRNA expression in LUAD was identified by the TCGA database. *p < 0.05, **p < 0.01, ***p < 0.001. Ctrl, control (untreated cells); DDP, cisplatin; Ine, inetetamab; GSDMB-N, GSDMB N-terminus; GSDMB-FL, full-length GSDMB

The NLR family member NOD-like receptor thermal protein domain associated protein 3 (NLRP3) can facilitate the formation of inflammasomes^43^. Pyroptosis is chiefly mediated through the activation of various caspases, including caspase-1, by the NLRP3 inflammasome^44^. ROS play a central role in NLRP3 inflammasome activation, thereby activating caspase-1 to induce pyroptosis^43^-^45^. Cisplatin is known to increase ROS levels in NSCLC cells^46^. Therefore, we speculated that the increased pyroptosis caused by the combination of cisplatin and inetetamab was most likely caused by the activation of NLRP3 in response to increased ROS levels. The determination of cellular ROS levels showed that inetetamab and cisplatin treatment elevated cellular ROS levels, while the increase was most pronounced in the combination treatment group (Fig. 6A). Moreover, acetyl cysteine (NAC), a ROS inhibitor, dramatically inhibited NLRP3 expression, GSDMB cleavage, and caspase-1 activation and attenuated the increase in the ratio of pyroptotic cells induced by the combined treatment in H1299, A549/DDP, and H1975 cells (Fig. 6B, C). Furthermore, the increase in NLRP3 expression was considerably more pronounced in the combination group than in the single-agent-treated groups (Fig. 6D). Therefore, we concluded that cisplatin combined with inetetamab induced NLRP3/caspase-1/GSDMB-mediated pyroptosis by triggering the generation of ROS to exert synergistic antitumor effects in LUAD cells.

Nuclear factor erythroid 2-like 2 (Nrf2), is one of the most essential antioxidant enzymes in modulating ROS^47^. Given the ample evidence that the downregulation of Nrf2 leads to ROS accumulation^48^, ^49^, Nrf2 protein levels were examined. Western blot analysis revealed that the combination therapy aggravated the inhibition of Nrf2 (Figure 6D). This is consistent with earlier studies showing that HER2-targeted drug therapy increases ROS levels by downregulating antioxidant enzymes^50^.

In addition, the ERK and AKT pathways are the major downstream pathways for HER2^51^, and Nrf2 is well regulated by PI3K/AKT^52^. We next examined the changes in ERK and AKT signaling to further investigate the specific molecular mechanism underlying the increase in pyroptosis induced by the combination of the two drugs. Western blot analysis showed that in H1299, A549/DDP, and H1975 cells, cisplatin treatment upregulated HER2 and downregulated p-AKT, whereas inetetamab decreased the expression of both HER2 and p-AKT. Interestingly, the levels of p-AKT were decreased following single-agent treatments and combined treatment, but the combination treatment exhibited the most pronounced suppression effect, which also directly suggests that the cotreatment should bemore advantageous (Fig. 6D). Sergina et al. reported compensatory feedback from AKT inhibition that resulted in elevated HER2 expression^53^, ^54^. Our data are generally consistent with their observations. There was a similar compensatory effect on HER2 when using cisplatin (Fig. 6D). However, inhibition of HER2 by inetetamab efficiently blocked this compensatory effect (Fig. 6D). Immunofluorescence experiments also demonstrated that following 48 hours of cisplatin exposure, HER2 expression significantly increased (Fig. 6E). After the addition of cisplatin, the HER2 expression level of A549/DDP cells was increased in a time- and dose-dependent manner (Fig. 6F, G). In addition, a positive correlation among HER2, AKT1/2, and Nrf2 was observed in the LUAD samples from the GEPIA database (Fig. 6H, I). The expression level of p-ERK was not different in the combination group compared with the single drug group (Fig. S4).

Collectively, these data showed that inetetamab enhanced the inhibitory effect of cisplatin on p-AKT, and this combination has an evident antitumor synergistic effect in LUAD cells. Our results also demonstrated that inetetamab blocked the compensatory negative-feedback loop caused by cisplatin treatment, thus providing a plausible molecular mechanism mediating the synergistic effect observed with inetetamab-cisplatin combined treatment. According to these experimental findings, inetetamab combined with cisplatin elevates ROS levels via the HER2/AKT/Nrf2 signaling pathway, which triggers NLRP3/caspase-1/GSDMB-mediated pyroptosis.

### Cisplatin enhances the PBMC-killing ability of inetetamab by inducing pyroptosis

**Figure 7:**
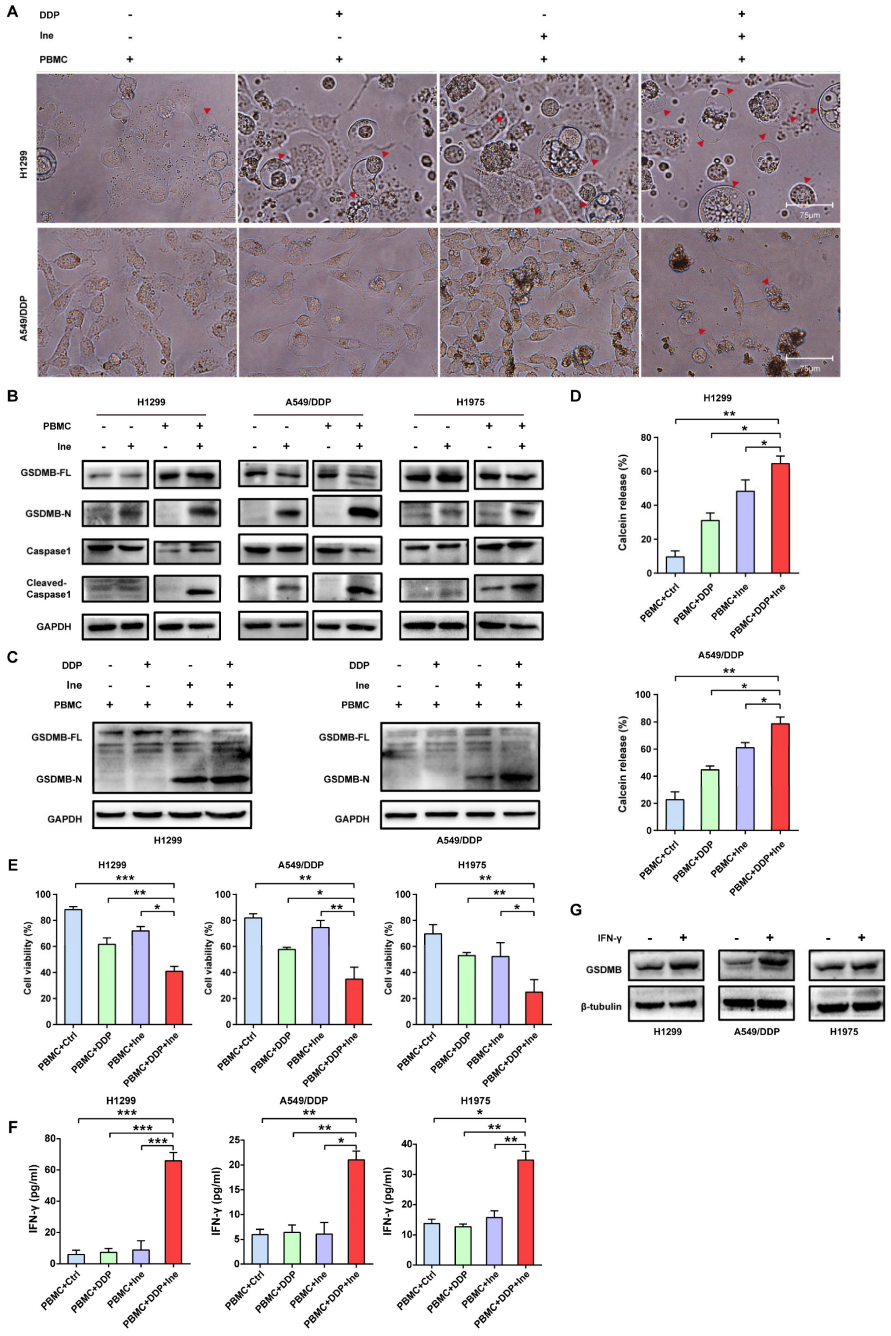
** Cisplatin enhances the PBMC-killing ability of inetetamab by inducing pyroptosis. (A)** Representative microscope capture of H1299 and A549/DDP cells treated with DDP or/and Ine for 4 days in the prescence of PBMCs. Red arrowheads indicated large bubbles emerging from the plasma membrane. Scale bar, 75 μm. **(B)** Western blot analysis of the cleavage of GSDMB and caspase-1 in H1299, A549/DDP and H1975 cells treated with Ine for 4 days with or without the prescence of PBMCs. **(C)** Western blot analysis of the cleavage of GSDMB in DDP, Ine and a combination of both treated H1299 and A549/DDP cells for 4 days in the prescence of PBMCs. GAPDH served as a loading control. **(D)** Calcein release in H1299 and A549/DDP treated with DDP, Ine and a combination of both for 4 days with the prescence of PBMCs. **(E)** Cell viability was measured through CCK-8 assay in indicated cells treated as described in C.** (F)** H1299, A549/DDP and H1975 cells were treated as described in C, and their supernatants were collected for ELISA to detect the level of IFN-γ secretion. **(G)** Western blot analysis of GSDMB expression in indicated cells with or without the pretreated of IFN-γ. β-tubulin served as a loading control. *p < 0.05, **p < 0.01, ***p < 0.001. Ctrl, control (untreated cells); DDP, cisplatin; Ine, inetetamab; GSDMB-N, GSDMB N-terminus; GSDMB-FL, full-length GSDMB

Inetetamab induces ADCC more potently than trastuzumab, thus facilitating greater antitumor effects in the presence of immune cells^16^, ^55^. Given that ADCC is a key mechanism for the antitumor activity of inetetamab^16^, ^55^, we need to consider the role of the interaction between inetetamab and immune cells in killing tumor cells in addition to the AKT/Nrf2/ROS/NLRP3/caspase-1/GSDMB axis when studying the mechanism by which cisplatin and inetetamab exert synergistic antitumor effects. NK cells are critical for ADCC^56^; however, the proportion of NK cells in PBMCs is only 5%-10, and PBMCs (not purified NK cells) are often used for ADCC assays^29^, ^57^. Subsequently, we explored the antitumor effects of cisplatin combined with inetetamab on H1299 and A549/DDP cells in the presence of PBMCs. Exposure to cisplatin at concentrations below 12.5 μM did not affect the cell viability of PBMCs, and the referenced dose is much higher than the concentration of cisplatin used in our experiments^11^. The addition of PBMCs increased the number of pyroptotic cells with characteristic large bubbles in the plasma membrane formed in the cisplatin or inetetamab groups, particularly in the inetetamab group, and this effect was more clearly observed in H1299 cells (Fig. 7A). Notably, the combined treatment group still showed the highest number of pyroptotic cells in the presence of PBMCs, which was more than that in the absence of PBMCs (Fig. 7A). Thus, Western blot analysis showed that inetetamab treatment activated caspase-1/GSDMB in the H1299, A549/DDP, and H1975 cell lines and promoted the release of cleaved caspase-1 and N-GSDMB in the presence of PBMCs (Fig. 7B). Moreover, the combined group still had the highest expression level of N-GSDMB, and single-agent inetetamab activated GSDMB more prominently than cisplatin because of the presence of PBMCs (Fig. 7C). Furthermore, the PBMC killing assays showed that the presence of PBMCs significantly enhanced the killing effect of the combined treatment on H1299 and A549/DDP cells compared with other groups (Fig. 7D), and the cell viability was evidently decreased (Fig. 7E).

Based on the above experimental results, we concluded that inetetamab combined with cisplatin promoted enlarged caspase-1/GSDMB-mediated pyroptosis in the presence of PBMCs.

IFN-γ released by activated cytotoxic lymphocytes triggers the upregulation of GSDMB, which promotes granzyme A-mediated pyroptotic killing of target cells^30^. In our work, IFN-γ in the supernatant of tumor cells cocultured with PBMCs in each group was measured using an ELISA technique. Cisplatin combined with inetetamab significantly increased the amount of IFN-γ secreted by PBMCs, as illustrated in Figure 7F. Elevation of IFN-γ levels may be one of the reasons for the increased pyroptosis of the combined group in the normal immune microenvironment. Herein, we further verified that IFN-γ increased the expression level of GSDMB in H1299, A549/DDP, and H1975 cells (Fig. 7G). In summary, IFN-γ induced signaling, which aided in GSDMB-mediated pyroptosis and contributed to the enhanced PBMC-killing effect of inetetamab combined with cisplatin in LUAD.

### Antitumor efficacy of cisplatin in combination with inetetamab in a xenograft model

**Figure 8:**
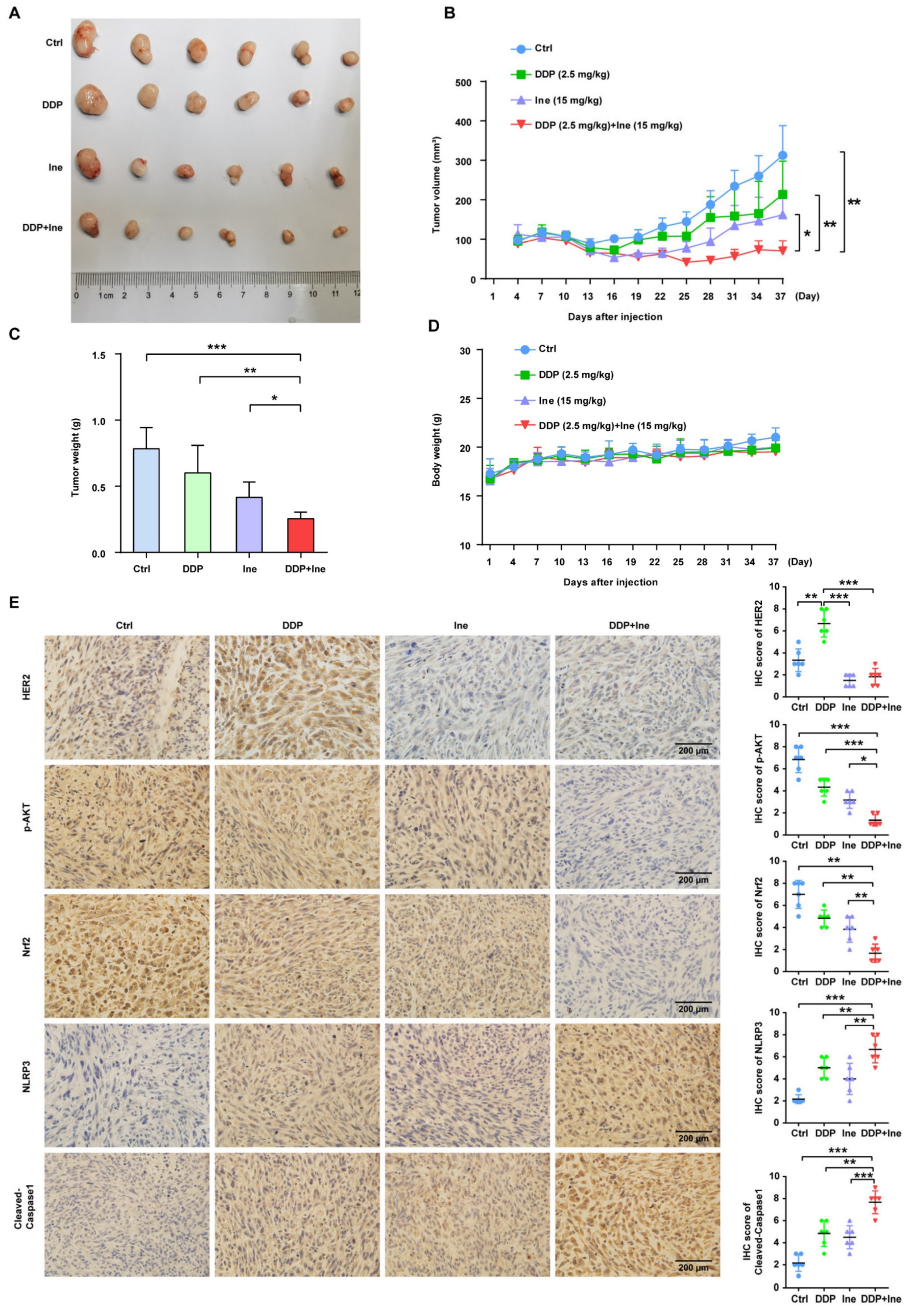
**Antitumor efficacy of inetetamab in combination with cisplatin in H1975 xenograft model *in vivo*. (A)** Representative images of tumors at 37 days after inoculation using H1975 cells treated with cisplatin (2.5 mg/kg weekly), inetetamab (15 mg/kg weekly), or their combination (2.5 mg/kg cisplatin +15 mg/kg inetetamab, weekly). The control group was injected with PBS. Administration of treatment began on day 7 after inoculation.** (B)** Tumor growth curves of the H1975 derived mouse xenograft study. The sizes of the tumors were measured every 3 days after inoculation (n = 6 for each experimental group). **(C)** Tumor weights were measured at day 37 after inoculation. **(D)** Body weights were recorded every 3 days after inoculation. **(E)** IHC analysis of HER2, p-AKT, Nrf2, NLRP3 and cleaved caspase-1 protein expression were performed using tumor sections of H1975 mouse xenografts treated as indicated above. Magnification, 400×; scale bar, 200µm. *p < 0.05, **p < 0.01, ***p < 0.001. Ctrl, control; DDP, cisplatin; Ine, inetetamab

Based on the synergistic inhibition of cisplatin and inetetamab combination treatment on LUAD cells *in vitro*, we estimated whether similar therapeutic effects could occur in a subcutaneous xenograft model. H1975 cells were subcutaneously injected into the right flanks of immunodeficient BALB/c nude mice, which harbored functional NK cells. After 30 days of treatment, we found that all treatment groups showed effective inhibition of tumor growth (Fig. 8A-C, Fig. S5). However, cisplatin combined with inetetamab treatment exhibited the greatest inhibitory effects on tumor volume and weight (Fig. 8A-C). Interestingly, inetetamab monotherapy was more effective than cisplatin monotherapy, which may be due to the ADCC effect exerted by inetetamab in addition to the triggering of pyroptosis *in vivo* (Fig. 8A-C). The body weight curves indicated that the combination did not appreciably contribute to *in vivo* systemic toxicity, as there were no significant changes in body weight between the combination-treated mice and the mice treated with inetetamab or cisplatin alone (Fig. 8D). At the molecular level, we used IHC assays for the mouse tumor tissues to test the expression status of NLRP3 and cleaved caspase-1 (pyroptosis related proteins), and found that the combined treatment induced a higher degree of pyroptosis than monotherapy (Fig. 8E). In addition, cisplatin treatment upregulated HER2 and downregulated p-AKT, whereas inetetamab treatment downregulated both HER2 and p-AKT compared to the control group (Fig. 8E). The lowest levels of p-AKT and Nrf2 and the highest levels of pyroptosis (NLRP3 and cleaved caspase-1) were observed in the combination group compared to other treatment groups (Fig. 8E). These data further validated that inetetamab and cisplatin synergistically enhance antitumor efficacy by inducing NLRP3/caspase-1-mediated pyroptosis by inhibiting HER2/AKT/Nrf2 signaling in mice bearing H1975 cell-derived tumors (Fig. 8E). In conclusion, these results suggested that the antitumor efficacy was stronger in the combined treatment group than in the groups treated with inetetamab or cisplatin alone *in vivo*.

## Discussion

**Figure 9:**
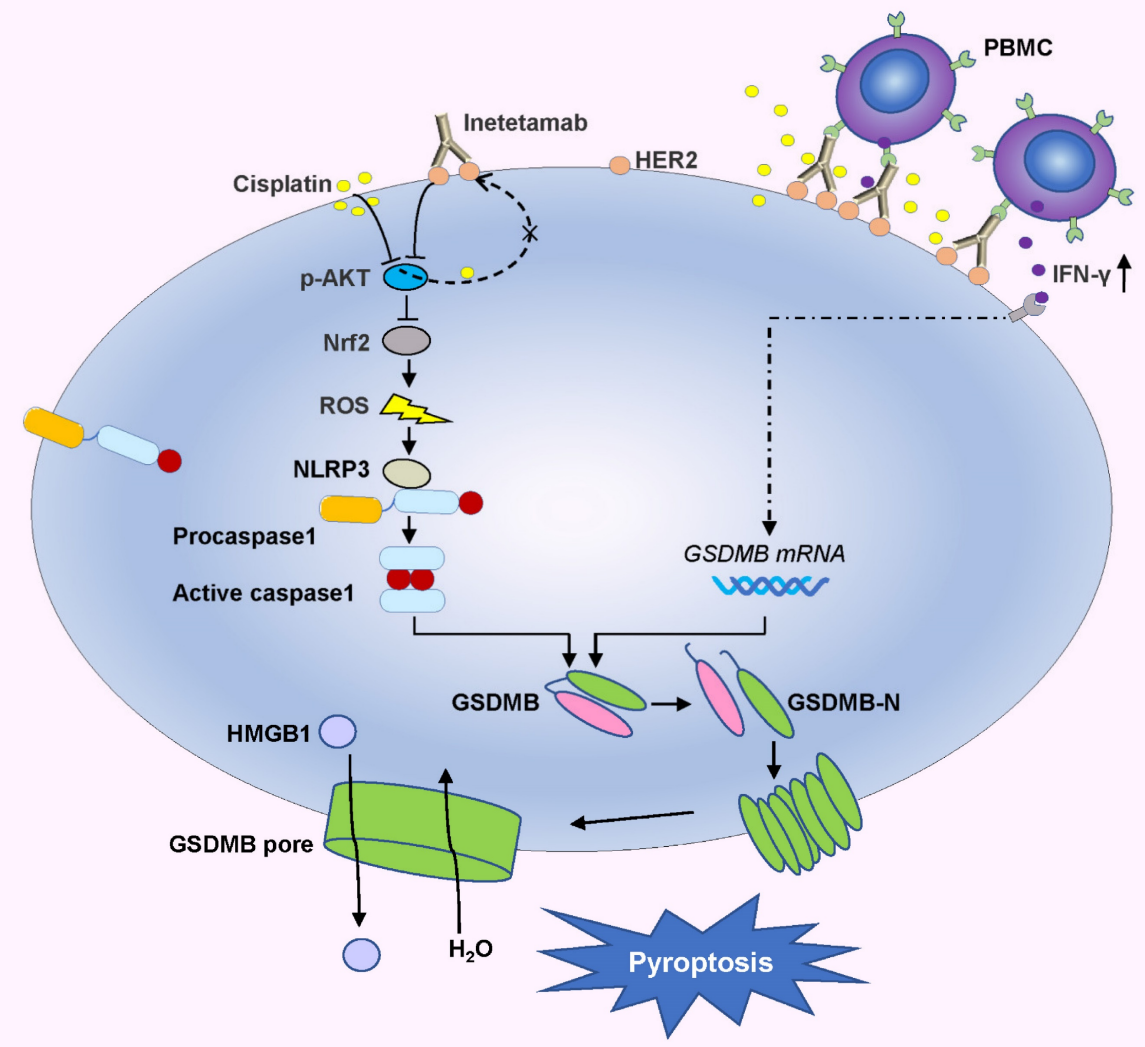
Inetetamab synergize with cisplatin inhibit HER2/AKT/Nrf2 signaling to elevate ROS levels, which proceeded to trigger NLRP3/caspase-1/GSDMB-mediated pyroptosis, to enhance the antitumor efficacy in LUAD cells. Furthermore, inetetamab combined with cisplatin enhanced PBMCs-killing ability by inducing pyroptosis, which can be explained by increased secretion of IFN-γ.

In the present investigation, we concluded that cisplatin and inetetamab exert synergistic antitumor effects on LUAD *in vitro* and *in vivo*. Our study showed that inetetamab synergized with cisplatin to inhibit HER2/AKT/Nrf2 signaling and elevate ROS levels, which triggered NLRP3/caspase-1/GSDMB-mediated pyroptosis to enhance antitumor efficacy in LUAD cells (Fig. 9). Furthermore, cisplatin greatly enhanced the PBMC-killing ability of inetetamab by inducing pyroptosis, which can be explained by increased secretion of IFN-γ (Fig. 9).

Pyroptosis is characterized by pore formation induced by the gasdermin family and subsequent cell swelling, lysis, and release of inflammatory factors as well as danger-associated molecular patterns (DAMPs), such as HMGB1^26^, ^58^, ^59^. Tumor cells that are more susceptible to pyroptosis are more sensitive to chemotherapy drugs^39^, ^60^, and chemotherapy can trigger pyroptosis in tumor cells^26^. Over the course of experiments, the anti-HER2 monoclonal antibody inetetamab combined with cisplatin led to an increase in the number of pyroptotic cells with typical bubbles emerging from the plasma membrane in LUAD cells; notably, pyroptosis is only one type of cell death among various forms. Therefore, we concluded that pyroptosis contributes to the synergistic antitumor effect of inetetamab and cisplatin in LUAD cells.

GSDMB acts as a tumor suppressor by triggering pyroptosis and promoting tumor clearance^30^; its role in cancer is gaining increased academic attention. Previously, GSDMB-related research has been more active in asthma, while our study fully characterizes GSDMB in patients with LUAD. Although it is well accepted that following activation by various inflammasomes, caspase-1 cleaves GSDMD^58^, our data showed that GSDMB is cleaved by activated caspase-1 to trigger pyroptosis in cells treated with cisplatin combined with inetetamab. Surprisingly, only recently has cleavage of GSDMB by caspase-1 been demonstrated by us and by others^61^. In addition, Panganiban et al.^42^ found that GSDMB is cleaved by caspase-1 at site 236 to induce pyroptosis in 293T cells. Recently, all gasdermins except DFNB59 were shown to possess intrinsic cytotoxic activity in their gasdermin-N domains, which is generally hidden by their gasdermin-C domains^59^. Although our data point to pyroptosis mediated by GSDMB, other GSDM-dependent pyroptosis cannot be ruled out as a potential mechanism underlying inetetamab and cisplatin synergy in LUAD. Moreover, cisplatin induced caspase-3 production and triggered GSDME-mediated pyroptosis^62^. However, whether GSDME-mediated pyroptosis plays a role in the synergistic antitumor effects of the two-drug combination requires further experimental validation.

HER2 is activated by homodimerization or by heterodimerization with other ErbB receptors and induces activation of AKT signaling pathways, thereby promoting cancer cell proliferation and survival^63^. AKT inhibition stimulates a compensatory increase in HER2 expression, which is linked to AKT-mediated negative feedback 54, 64. In the present report, we observed that AKT inhibition induced by cisplatin treatment also promoted negative feedback that was manifested by an increase in HER2 expression in LUAD cells. When cisplatin is given together with inetetamab, the feedback loop fails because of the disruption of HER2 homodimerization or heterodimerization, which may be the mechanism for the synergistic antitumor effects. However, the exact mechanism deserves further investigation. Moreover, the relationship between HER2/AKT/Nrf2/ROS is well established, as is the relationship between ROS/NLRP3/caspase-1^65^, ^66^. Recently, some researchers have indicated that coregulatory roles of HER2/HER3, Nrf2, and ROS may exist in several types of cancers including breast and ovarian cancers^65^, ^66^. Belmonte et al. found that HER2 overexpression upregulates antioxidant signaling and reduces the basal level of ROS in various tumors^50^. Valentina et al. found that Nrf2 could be a potential effector of resistance to trastuzumab in gastric cancer through the PI3K/AKT/mTOR/RPS6 pathway^67^. Hence, our data showed that the combination therapy exacerbated not only the inhibition of p-AKT but also the inhibition of Nrf2, which caused further accumulation of cellular ROS. Furthermore, in our experiments, ROS scavengers were sufficient to reverse NLRP3/caspase-1/GSDMB-mediated pyroptosis of LUAD induced by cisplatin + inetetamab. Considering these data, we concluded that cisplatin + inetetamab-induced pyroptosis in LUAD cells is regulated by the HER2/AKT/Nrf2/ROS/NLRP3/caspase-1/GSDMB signaling pathway.

ADCC, one of the important mechanisms for the antitumor activity of inetetamab, is initiated when the FCγ receptor on natural killer cells (NK) binds to the Fc portion of inetetamab. The combined use of trastuzumab and erlotinib enhanced the ADCC of wild-type erlotinib-sensitive NSCLC cell lines^68^. Naruse et al. reported that trastuzumab combined with cisplatin was more cytotoxic to tumor cells, including NSCLC cells, in the presence of PBMCs^11^. Similarly, in our study, we found that inetetamab combined with cisplatin enhanced PBMC-killing ability by inducing enlarged pyroptosis. Moreover, cisplatin combined with inetetamab can significantly increase the amount of IFN-γ secreted by PBMCs. IFN-γ was recently described to markedly upregulate GSDMB^30^, which was further confirmed. In addition, IFN-γ alone was confirmed to promote cell cytotoxicity and robustly induce caspase-1 expression^69^. Based on our experimental data and literature reports, we concluded that the increased secretion of IFN-γ, which contributes to GSDMB-mediated pyroptosis, may be responsible for the significant enhancement of PBMC-killing ability by cisplatin combined with inetetamab. However, detailed mechanistic studies are still needed to identify which types of immune cells play a major role. Considering that NK cells are the main effectors of ADCC, we hypothesized that they might be involved in the enhanced PBMC-killing ability induced by combination therapy. This is consistent with the role of NK cells as major producers of IFN-γ within the PBMC population^70^. However, further studies are needed to confirm the important role of NK cells in the combination of the two drugs.

Cisplatin-based chemotherapy is the most common therapy for LUAD cancer^2^; however, its efficacy is greatly limited because of drug resistance^3^. In our study, adding inetetamab to cisplatin enhanced the antitumor effect not only in cisplatin-sensitive cell lines (H1299, H1975) but also in cisplatin-resistant cell lines (A549/DDP). Considering the complex and multifactorial mechanism^4^, ^5^, resistance to cisplatin in LUAD is still an intractable issue in the clinic. Hence, our findings provide a therapeutic reference for patients with HER2-positive lung adenocarcinoma, not only by reducing the dosage of cisplatin, which leads to fewer toxic side effects of chemotherapy but also by reducing the financial burden. Our findings undoubtedly provide a promising therapeutic strategy to overcome cisplatin resistance in LUAD.

Nevertheless, our study only revealed one of the possible mechanisms. The exact molecular mechanism of the synergistic antitumor effect of inetetamab combined with cisplatin, which involves triggering of pyroptosis, needs further investigation. In addition, all the mechanisms attributed to inetetamab may work for trastuzumab too, and whether the synergistic effect of inetetamab in combination with cisplatin is better than that of trastuzumab in combination with cisplatin in LUAD needs to be further verified.

## Conclusion

Our findings prove for the first time that inetetamab combined with cisplatin inhibits HER2/AKT/Nrf2 signaling to increase ROS levels, which triggers NLRP3/caspase-1/GSDMB-mediated pyroptosis to enhance antitumor efficacy. Furthermore, inetetamab combined with cisplatin enhanced the PBMC-killing ability by inducing pyroptosis. We also found that the combination treatment exhibited the greatest antitumor effect *in vivo*. Our study reveals that the anti-HER2 monoclonal antibody inetetamab may be an attractive candidate for LUAD therapy.

## Supplementary Material

Supplementary figures.

## References

[1] Tammela T, Sanchez-Rivera FJ, Cetinbas NM, Wu K, Joshi NS, Helenius K (2017). A Wnt-producing niche drives proliferative potential and progression in lung adenocarcinoma. Nature.

[2] Heigener DF, Kerr KM, Laing GM, Mok TSK, Moiseyenko FV, Reck M (2019). Redefining Treatment Paradigms in First-line Advanced Non-Small-Cell Lung Cancer. Clin Cancer Res.

[3] El-Hussein A, Manoto SL, Ombinda-Lemboumba S, Alrowaili ZA, Mthunzi-Kufa P (2021). A Review of Chemotherapy and Photodynamic Therapy for Lung Cancer Treatment. Anticancer Agents Med Chem.

[4] Yu WK, Wang Z, Fong CC, Liu D, Yip TC, Au SK (2017). Chemoresistant lung cancer stem cells display high DNA repair capability to remove cisplatin-induced DNA damage. Br J Pharmacol.

[5] Makovec T (2019). Cisplatin and beyond: molecular mechanisms of action and drug resistance development in cancer chemotherapy. Radiol Oncol.

[6] Kryczka J, Kryczka J, Czarnecka-Chrebelska KH, Brzezianska-Lasota E (2021). Molecular Mechanisms of Chemoresistance Induced by Cisplatin in NSCLC Cancer Therapy. Int J Mol Sci.

[7] Kim ES, Lee JJ, He G, Chow CW, Fujimoto J, Kalhor N (2012). Tissue platinum concentration and tumor response in non-small-cell lung cancer. J Clin Oncol.

[8] Riudavets M, Sullivan I, Abdayem P, Planchard D (2021). Targeting HER2 in non-small-cell lung cancer (NSCLC): a glimpse of hope? An updated review on therapeutic strategies in NSCLC harbouring HER2 alterations. ESMO Open.

[9] Oh DY, Bang YJ (2020). HER2-targeted therapies - a role beyond breast cancer. Nat Rev Clin Oncol.

[10] Kulhari H, Pooja D, Rompicharla SV, Sistla R, Adams DJ (2015). Biomedical applications of trastuzumab: as a therapeutic agent and a targeting ligand. Med Res Rev.

[11] Naruse I, Fukumoto H, Saijo N, Nishio K (2002). Enhanced anti-tumor effect of trastuzumab in combination with cisplatin. Jpn J Cancer Res.

[12] Hong JH, Tong ZJ, Wei TE, Lu YC, Huang CY, Huang CY (2022). Cigarette Smoke Containing Acrolein Contributes to Cisplatin Resistance in Human Bladder Cancers through the Regulation of HER2 Pathway or FGFR3 Pathway. Mol Cancer Ther.

[13] Udpa N, Million RP (2016). Monoclonal antibody biosimilars. Nat Rev Drug Discov.

[14] Derakhshani A, Rezaei Z, Safarpour H, Sabri M, Mir A, Sanati MA (2020). Overcoming trastuzumab resistance in HER2-positive breast cancer using combination therapy. J Cell Physiol.

[15] Waller CF, Mobius J, Fuentes-Alburo A (2021). Intravenous and subcutaneous formulations of trastuzumab, and trastuzumab biosimilars: implications for clinical practice. Br J Cancer.

[16] Zhou X, Yu J, Wang W, Song G, Wang X, Ren J (2015). A phase I dose-escalation study of a biosimilar trastuzumab in Chinese metastasis breast cancer patients. Springerplus.

[17] Tang Z, Li C, Kang B, Gao G, Li C, Zhang Z (2017). GEPIA: a web server for cancer and normal gene expression profiling and interactive analyses. Nucleic Acids Res.

[18] Cao L, Wang F, Li S, Wang X, Huang D, Jiang R (2019). PIM1 kinase promotes cell proliferation, metastasis and tumor growth of lung adenocarcinoma by potentiating the c-MET signaling pathway. Cancer Lett.

[19] Liu D, Lin L, Wang Y, Chen L, He Y, Luo Y (2020). PNO1, which is negatively regulated by miR-340-5p, promotes lung adenocarcinoma progression through Notch signaling pathway. Oncogenesis.

[20] Xing Y, Liu Y, Liu T, Meng Q, Lu H, Liu W (2018). TNFAIP8 promotes the proliferation and cisplatin chemoresistance of non-small cell lung cancer through MDM2/p53 pathway. Cell Commun Signal.

[21] Uphoff CC, Drexler HG (2011). Detecting mycoplasma contamination in cell cultures by polymerase chain reaction. Methods Mol Biol.

[22] Ai M, Qiu S, Lu Y, Fan Z (2013). HER2 regulates Brk/PTK6 stability via upregulating calpastatin, an inhibitor of calpain. Cell Signal.

[23] He Y, Xiao M, Fu H, Chen L, Qi L, Liu D (2020). cPLA2alpha reversibly regulates different subsets of cancer stem cells transformation in cervical cancer. Stem Cells.

[24] Chou TC, Talalay P (1984). Quantitative analysis of dose-effect relationships: the combined effects of multiple drugs or enzyme inhibitors. Adv Enzyme Regul.

[25] Li L, Song D, Qi L, Jiang M, Wu Y, Gan J (2021). Photodynamic therapy induces human esophageal carcinoma cell pyroptosis by targeting the PKM2/caspase-8/caspase-3/GSDME axis. Cancer Lett.

[26] Wang Y, Gao W, Shi X, Ding J, Liu W, He H (2017). Chemotherapy drugs induce pyroptosis through caspase-3 cleavage of a gasdermin. Nature.

[27] Liu L, Geng H, Mei C, Chen L (2021). Zoledronic Acid Enhanced the Antitumor Effect of Cisplatin on Orthotopic Osteosarcoma by ROS-PI3K/AKT Signaling and Attenuated Osteolysis. Oxid Med Cell Longev.

[28] Xu H, Li D, Ma J, Zhao Y, Xu L, Tian R (2021). The IL-33/ST2 axis affects tumor growth by regulating mitophagy in macrophages and reprogramming their polarization. Cancer Biol Med.

[29] Chew HY, De Lima PO, Gonzalez Cruz JL, Banushi B, Echejoh G, Hu L (2020). Endocytosis Inhibition in Humans to Improve Responses to ADCC-Mediating Antibodies. Cell.

[30] Zhou Z, He H, Wang K, Shi X, Wang Y, Su Y (2020). Granzyme A from cytotoxic lymphocytes cleaves GSDMB to trigger pyroptosis in target cells. Science.

[31] Hou J, Zhao R, Xia W, Chang CW, You Y, Hsu JM (2020). PD-L1-mediated gasdermin C expression switches apoptosis to pyroptosis in cancer cells and facilitates tumour necrosis. Nat Cell Biol.

[32] Chen X, Chen H, Yao H, Zhao K, Zhang Y, He D (2021). Turning up the heat on non-immunoreactive tumors: pyroptosis influences the tumor immune microenvironment in bladder cancer. Oncogene.

[33] Cui J, Song Y, Han X, Hu J, Chen Y, Chen X (2020). Targeting 14-3-3zeta Overcomes Resistance to Epidermal Growth Factor Receptor-Tyrosine Kinase Inhibitors in Lung Adenocarcinoma via BMP2/Smad/ID1 Signaling. Front Oncol.

[34] Shen SM, Zhang C, Ge MK, Dong SS, Xia L, He P (2019). PTENalpha and PTENbeta promote carcinogenesis through WDR5 and H3K4 trimethylation. Nat Cell Biol.

[35] Zhang S, Cao M, Yan S, Liu Y, Fan W, Cui Y (2022). TRIM44 promotes BRCA1 functions in HR repair to induce Cisplatin Chemoresistance in Lung Adenocarcinoma by Deubiquitinating FLNA. Int J Biol Sci.

[36] Kosciuczuk EM, Saleiro D, Kroczynska B, Beauchamp EM, Eckerdt F, Blyth GT (2016). Merestinib blocks Mnk kinase activity in acute myeloid leukemia progenitors and exhibits antileukemic effects in vitro and in vivo. Blood.

[37] Villar-Quiles RN, Catervi F, Cabet E, Juntas-Morales R, Genetti CA, Gidaro T (2020). ASC-1 Is a Cell Cycle Regulator Associated with Severe and Mild Forms of Myopathy. Ann Neurol.

[38] Wang Q, Wang Y, Ding J, Wang C, Zhou X, Gao W (2020). A bioorthogonal system reveals antitumour immune function of pyroptosis. Nature.

[39] Peng Z, Wang P, Song W, Yao Q, Li Y, Liu L (2020). GSDME enhances Cisplatin sensitivity to regress non-small cell lung carcinoma by mediating pyroptosis to trigger antitumor immunocyte infiltration. Signal Transduct Target Ther.

[40] Ding J, Wang K, Liu W, She Y, Sun Q, Shi J (2016). Pore-forming activity and structural autoinhibition of the gasdermin family. Nature.

[41] Martinez-Garcia JJ, Martinez-Banaclocha H, Angosto-Bazarra D, de Torre-Minguela C, Baroja-Mazo A, Alarcon-Vila C (2019). P2X7 receptor induces mitochondrial failure in monocytes and compromises NLRP3 inflammasome activation during sepsis. Nat Commun.

[42] Panganiban RA, Sun M, Dahlin A, Park HR, Kan M, Himes BE (2018). A functional splice variant associated with decreased asthma risk abolishes the ability of gasdermin B to induce epithelial cell pyroptosis. J Allergy Clin Immunol.

[43] Barry R, John SW, Liccardi G, Tenev T, Jaco I, Chen CH (2018). SUMO-mediated regulation of NLRP3 modulates inflammasome activity. Nat Commun.

[44] Yu W, Zong S, Zhou P, Wei J, Wang E, Ming R (2022). Cochlear Marginal Cell Pyroptosis Is Induced by Cisplatin via NLRP3 Inflammasome Activation. Front Immunol.

[45] Tang D, Kang R, Berghe TV, Vandenabeele P, Kroemer G (2019). The molecular machinery of regulated cell death. Cell Res.

[46] Huang S, He T, Yang S, Sheng H, Tang X, Bao F (2020). Metformin reverses chemoresistance in non-small cell lung cancer via accelerating ubiquitination-mediated degradation of Nrf2. Transl Lung Cancer Res.

[47] Ying S, Tan M, Feng G, Kuang Y, Chen D, Li J (2020). Low-intensity Pulsed Ultrasound regulates alveolar bone homeostasis in experimental Periodontitis by diminishing Oxidative Stress. Theranostics.

[48] Chang CW, Chen YS, Tsay YG, Han CL, Chen YJ, Yang CC (2018). ROS-independent ER stress-mediated NRF2 activation promotes warburg effect to maintain stemness-associated properties of cancer-initiating cells. Cell Death Dis.

[49] Lee BWL, Ghode P, Ong DST (2019). Redox regulation of cell state and fate. Redox Biol.

[50] Belmonte F, Das S, Sysa-Shah P, Sivakumaran V, Stanley B, Guo X (2015). ErbB2 overexpression upregulates antioxidant enzymes, reduces basal levels of reactive oxygen species, and protects against doxorubicin cardiotoxicity. Am J Physiol Heart Circ Physiol.

[51] Smith AE, Ferraro E, Safonov A, Morales CB, Lahuerta EJA, Li Q (2021). HER2 + breast cancers evade anti-HER2 therapy via a switch in driver pathway. Nat Commun.

[52] Bai X, Gou X, Cai P, Xu C, Cao L, Zhao Z (2019). Sesamin Enhances Nrf2-Mediated Protective Defense against Oxidative Stress and Inflammation in Colitis via AKT and ERK Activation. Oxid Med Cell Longev.

[53] Chandarlapaty S, Sawai A, Scaltriti M, Rodrik-Outmezguine V, Grbovic-Huezo O, Serra V (2011). AKT inhibition relieves feedback suppression of receptor tyrosine kinase expression and activity. Cancer Cell.

[54] Sergina NV, Rausch M, Wang D, Blair J, Hann B, Shokat KM (2007). Escape from HER-family tyrosine kinase inhibitor therapy by the kinase-inactive HER3. Nature.

[55] Zhang X, Chen J, Weng Z, Li Q, Zhao L, Yu N (2020). A new anti-HER2 antibody that enhances the anti-tumor efficacy of trastuzumab and pertuzumab with a distinct mechanism of action. Mol Immunol.

[56] Wu HT, Zhao XY (2022). Regulation of CD38 on Multiple Myeloma and NK Cells by Monoclonal Antibodies. Int J Biol Sci.

[57] Park JE, Kim SE, Keam B, Park HR, Kim S, Kim M (2020). Anti-tumor effects of NK cells and anti-PD-L1 antibody with antibody-dependent cellular cytotoxicity in PD-L1-positive cancer cell lines. J Immunother Cancer.

[58] Wang K, Sun Q, Zhong X, Zeng M, Zeng H, Shi X (2020). Structural Mechanism for GSDMD Targeting by Autoprocessed Caspases in Pyroptosis. Cell.

[59] Fang Y, Tian S, Pan Y, Li W, Wang Q, Tang Y (2020). Pyroptosis: A new frontier in cancer. Biomed Pharmacother.

[60] Deng BB, Jiao BP, Liu YJ, Li YR, Wang GJ (2020). BIX-01294 enhanced chemotherapy effect in gastric cancer by inducing GSDME-mediated pyroptosis. Cell Biol Int.

[61] Li L, Li Y, Bai Y (2020). Role of GSDMB in Pyroptosis and Cancer. Cancer Manag Res.

[62] Zhang Z, Zhang Y, Xia S, Kong Q, Li S, Liu X (2020). Gasdermin E suppresses tumour growth by activating anti-tumour immunity. Nature.

[63] Weinberg F, Peckys DB, de Jonge N (2020). EGFR Expression in HER2-Driven Breast Cancer Cells. Int J Mol Sci.

[64] Junttila TT, Akita RW, Parsons K, Fields C, Lewis Phillips GD, Friedman LS (2009). Ligand-independent HER2/HER3/PI3K complex is disrupted by trastuzumab and is effectively inhibited by the PI3K inhibitor GDC-0941. Cancer Cell.

[65] Yang Y, Tian Z, Guo R, Ren F (2020). Nrf2 Inhibitor, Brusatol in Combination with Trastuzumab Exerts Synergistic Antitumor Activity in HER2-Positive Cancers by Inhibiting Nrf2/HO-1 and HER2-AKT/ERK1/2 Pathways. Oxid Med Cell Longev.

[66] Manandhar S, Choi BH, Jung KA, Ryoo IG, Song M, Kang SJ (2012). NRF2 inhibition represses ErbB2 signaling in ovarian carcinoma cells: implications for tumor growth retardation and docetaxel sensitivity. Free Radic Biol Med.

[67] Gambardella V, Gimeno-Valiente F, Tarazona N, Martinez-Ciarpaglini C, Roda D, Fleitas T (2019). NRF2 through RPS6 Activation Is Related to Anti-HER2 Drug Resistance in HER2-Amplified Gastric Cancer. Clin Cancer Res.

[68] Cavazzoni A, Alfieri RR, Cretella D, Saccani F, Ampollini L, Galetti M (2012). Combined use of anti-ErbB monoclonal antibodies and erlotinib enhances antibody-dependent cellular cytotoxicity of wild-type erlotinib-sensitive NSCLC cell lines. Mol Cancer.

[69] Detjen KM, Farwig K, Welzel M, Wiedenmann B, Rosewicz S (2001). Interferon gamma inhibits growth of human pancreatic carcinoma cells via caspase-1 dependent induction of apoptosis. Gut.

[70] Swaim CD, Scott AF, Canadeo LA, Huibregtse JM (2017). Extracellular ISG15 Signals Cytokine Secretion through the LFA-1 Integrin Receptor. Mol Cell.

