# Supplementary material for: Inetetamab, a novel anti-HER2 monoclonal antibody, exhibits potent synergistic anticancer effects with cisplatin by inducing pyroptosis in lung adenocarcinoma
Source: Int J Biol Sci. 2023 Aug 6;19(13):4061–81. doi: 10.7150/ijbs.82980 (PMC10496496; doi:10.7150/ijbs.82980)
Supplement: Supplementary file 1 — Supplementary figures. [file ijbsv19p4061s1.pdf]

Supplementary Material

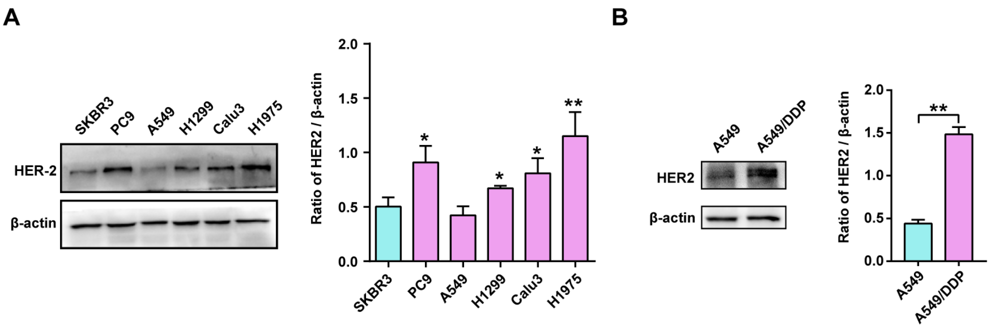

Supplementary Figure 1. (A) HER2 expression levels in SKBR3, PC9, A549, H1299, Calu3 and H1975 were examined by Western blotting. β-actin was used as a loading control. **(B)** HER2 expression levels in A549 and A549/DDP were examined by Western blotting. β-actin was used as a loading control. **p* < 0.05, ***p* < 0.01

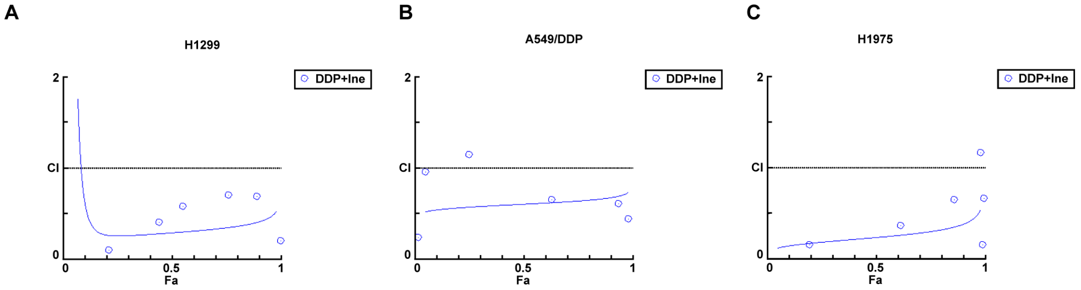

Supplementary Figure 2. (A) H1299 cells were treated with different combinations of concentrations of DDP and Ine for 5 days, and the CI values of DDP combined with Ine were calculated for H1299 cell line. **(B)** A549/DDP cells were treated with different combinations of concentrations of DDP and Ine for 5 days, and the CI values of DDP combined with Ine were calculated for A549/DDP cell line. **(C)** H1975 cells were treated with different combinations of concentrations of DDP and Ine for 5 days, and the CI values of DDP combined with Ine were calculated for H1975 cell line. Ctrl, control (untreated cells); DDP, cisplatin; Ine, inetetamab

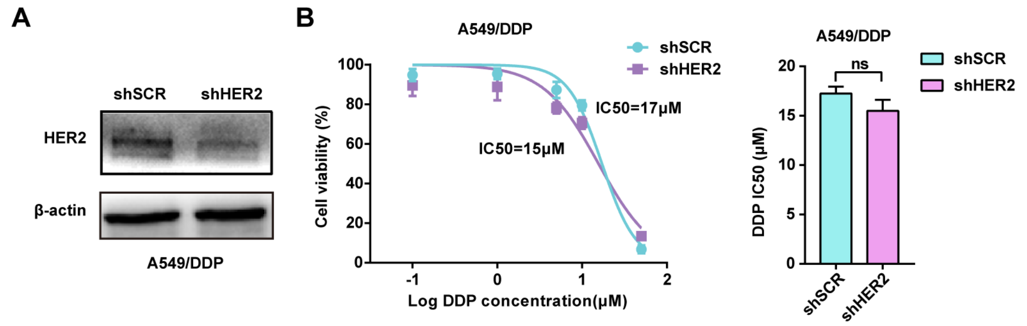

Supplementary Figure 3. (A) The levels of HER2 protein in A549/DDP-SCR and A549/DDP-shHER2 cells were examined by Western blot. **(B)** A549/DDP-shHER2 and corresponding vector control cells were treated with the indicated doses of cisplatin for 48 h, and cell viability was analyzed by a CCK-8 assay. ns, not statistically significant.

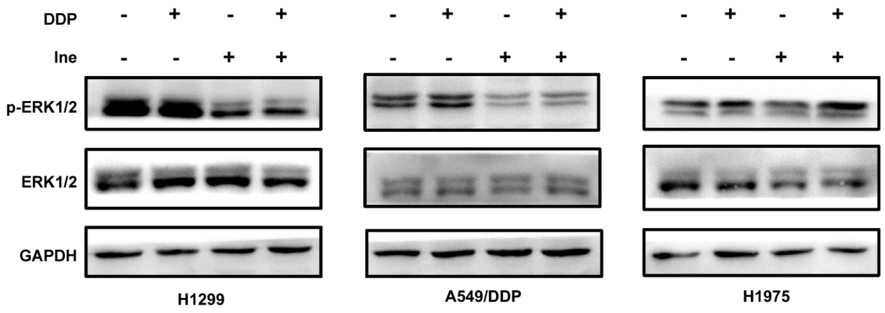

Supplementary Figure 4. Western blot analysis of p-ERK1/2 and ERK1/2 proteins levels in indicated cells treated with DDP and Ine alone or in combination for 4 days. GAPDH served as a loading control. Ine, inetetamab; DDP, cisplatin

Ctrl

DDP

Ine

DDP+Ine

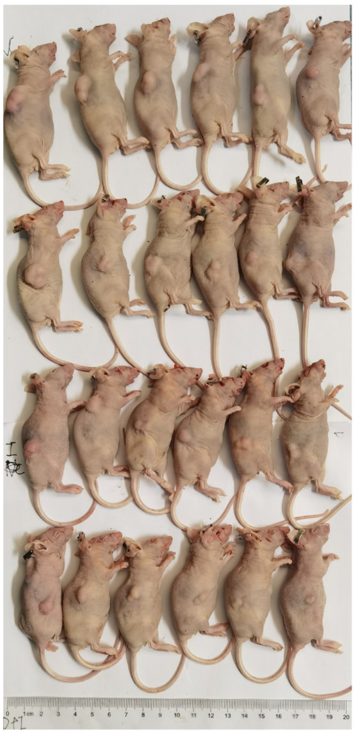

Supplementary Figure 5. H1975 cells were subcutaneously injected to the right flanks of BALB/c nude mice. The mice were treated with inetetamab, cisplatin, or their combination as described in Methods. The control group was injected with PBS.
